# Th17 cells favor migration and invasiveness of cervical cancer cells under hypoxia in an IGF2BP2‐dependent manner

**DOI:** 10.1002/ijc.70340

**Published:** 2026-01-24

**Authors:** Selina Gies, Maike Pohlers, Tanja Tänzer, Emmanuel Ampofo, Matthias W. Laschke, Moritz Schäfer, Yoo‐Jin Kim, Rainer Maria Bohle, Erich‐Franz Solomayer, Konrad Wagner, Martin Empting, Alexandra K. Kiemer, Barbara Walch‐Rückheim

**Affiliations:** ^1^ Experimental Gynecological Oncology, Gynecology, Faculty of Medicine University of Augsburg Augsburg Germany; ^2^ Center of Human and Molecular Biology (ZHMB), Institute of Virology Saarland University Homburg Germany; ^3^ Institute for Clinical and Experimental Surgery Saarland University Homburg Germany; ^4^ PharmaScienceHub (PSH) Saarbrücken Germany; ^5^ Center for Gender‐Specific Biology and Medicine (CGBM) Saarland University Saarbrücken Germany; ^6^ Institute of Pathology, Saarland University Medical Center Homburg Germany; ^7^ Department of Obstetrics and Gynecology, Saarland University Medical Center Homburg Germany; ^8^ Department of Pharmacy Saarland University Saarbrücken Germany

**Keywords:** cervical cancer progression, hypoxia, IGF2BP2, RNA binding protein (RBP), Th17 cells

## Abstract

Hypoxic regions of cervical cancers mediate suppression of the human papillomavirus (HPV) oncoproteins E6 and E7 in an AKT‐dependent manner causing oxygen‐dependent reversible growth arrest of cancer cells. Furthermore, numbers of T‐helper (Th)‐17 cells increase during cervical carcinogenesis in cancer tissues, Th17 differentiation is favored by hypoxia and their presence is linked with AKT‐dependent therapy resistance, metastases and relapse. As both factors, hypoxia and Th17 cells, are associated with poor prognosis of patients, potential synergistic mechanisms between both are not described so far. In this study, we showed that Th17 cells enhance the expression of hypoxia‐related glycolytic enzymes and transporters and functionally favor increased glucose uptake, proliferation and migration of 2D cultures of hypoxic cervical cancer cells as well as invasion of 3D spheroids. As the responsible mediator of increased proliferation, migration and invasion, we identified the RNA‐binding protein IGF2BP2 by using small interfering RNAs (siRNAs) for IGF2BP2 as well as small molecule IGF2BP2 inhibitors of the benzamidobenzoic acid class. Consistently, Th17 numbers in cervical cancer biopsies correlated with IGF2BP2 expression associated with lymph node metastases and relapse. Correspondingly, a combination of IGF2BP2 expression and Th17 cell numbers >10/mm^2^ in situ was associated with reduced recurrence‐free survival. In summary, we unraveled a previously unknown molecular mechanism by which Th17 cells promote tumor progression under hypoxic conditions and suggest evaluation of Th17 cells as well as IGF2BP2 as potential target for therapeutic approaches in cervical cancer.

## Graphical abstract

What's new?

Hypoxic regions and inflammatory Th17 cells in the tumor environment are both associated with poor prognosis in cervical cancer. However, synergistic mechanisms between hypoxia and Th17 cells remain elusive. This study demonstrated Th17–hypoxia‐driven mechanisms underlying cervical cancer progression. The authors identified Th17–hypoxia interactions promoting the proliferation, migration, and invasion of hypoxic cancer cells, with RNA‐binding protein IGF2BP2 as the responsible mediator. Moreover, high numbers of Th17 cells combined with high IGF2BP2 levels in situ were associated with cancer progression, highlighting Th17 cells as well as IGF2BP2 proteins as potential targets for (immuno)therapy.

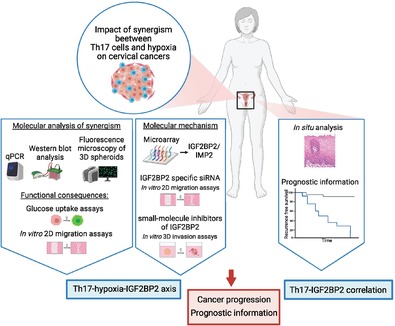

**Abbreviations**
AKT: protein kinase B
BSA: bovine serum albumin
CAIX: carbonic anhydrase IX
CD: cluster of differentiation
CM: conditioned media
CRT: chemoradiotherapy
DAB: 3,3′‐diaminobenzidine
DAPI: 4′,6′‐diamidin‐2‐phenylindole
DMEM: Dulbecco's modified eagle medium
DMSO: dimethyl sulfoxide
ELISA: enzyme‐linked immunosorbent assay
EMT: epithelial‐to‐mesenchymal transition
FCS: fetal calf serum
FIGO: international federation of gynecology and obstetrics
HIF1A: hypoxia inducible factor 1 subunit alpha
HPV: human papillomavirus
IF: immunofluorescence
IGF2BP2: insulin‐like growth factor 2 mRNA‐binding protein 2
IHC: immunohistochemistry
IL: interleukin
IRS: immunoreactive score
mTORC2: mammalian target of rapamycin complex 1
NBDG: 2‐(N‐[7‐nitrobenz‐2‐oxa‐1,3‐diazol‐4‐yl]amino)‐2‐deoxyglucose
PBS: phosphate buffered saline
PCR: polymerase chain reaction
PFA: paraformaldehyde
PI3K: phosphoinositide 3‐kinases
POX: peroxidase
RFS: recurrence‐free survival
ROC: receiver operator characteristics
RPMI: Roswell Park Memorial Institute
SCC: squamous cell carcinomas
siRNA: small interfering RNAs
SLC2A1: solute carrier family 2 member 1
TBS: tris‐buffered saline
TE: tris EDTA
Th: T helper cells

## 1 INTRODUCTION

The prognosis for patients with advanced, metastatic cervical cancers remains poor. Cervical cancer is the fourth most common cancer in women worldwide with 661,021 new cases and 348,189 cancer‐related deaths in 2022.^1^ Cervical carcinogenesis is a multistep process including precancerous lesions driven by persistent infection of high‐risk human papillomaviruses (HPV).^2^ Cervical cancers are staged according to the International Federation of Gynecology and Obstetrics (FIGO) classification representing the basis for the guideline‐based stage‐dependent therapy. Treatment approaches include, besides surgery alone in early stages, adjuvant platinum‐based concurrent chemoradiotherapy (CRT) for advanced cancers and primary CRT for locally advanced often inoperable cervical carcinoma (FIGO > IIB).^3^ However, the initial tumor stage critically influences the course of disease within the first 2 years after completing primary treatment, resulting in 5‐year survival ranging from over 90% if diagnosed in an early, localized stage to less than 20% if cervical cancer is diagnosed as distant or metastatic.^4^ Thus, there is a need to clarify molecular mechanisms driving cancer progression as the basis of novel (immuno)therapeutic therapies.

Solid cancers exhibit regions with low oxygen concentrations (<1.5% O_2_) termed hypoxic regions^5^ whose presence was linked with tumor development and progression.^6^ Clinically, hypoxic tumors are associated with resistance toward chemo‐ and radiotherapy and considered a negative prognostic marker for many cancers, including HPV‐positive tumors.^7^, ^8^ Cervical cancers comprise more‐ and less‐oxygenated regions, and the median O_2_ concentration is indicated with 1.2%^7^ in comparison to healthy cervical tissue stated with 5.5% O_2_.^9^ Interestingly, hypoxia activates the PI3K/mTORC2/AKT signaling pathway in cervical cancer cells responsible for hypoxia‐mediated downregulation of the expression of the viral early proteins E6 and E7.^10^ The presence of these two HPV oncoproteins is linked to sustained proliferation of HPV‐positive tumors.^11^, ^12^ Consequently, hypoxia‐induced repression of E6 and E7 enables hypoxic cervical cancer cells to enter a dormant state, potentially linked with therapy resistance and tumor recurrence, and characterized by a reversible growth arrest abolishable upon reoxygenation.^5^

Besides the role of hypoxia, the composition of the micromilieu is crucial for tumor development and therapy responses and affects the progression of disease.^13^ Strong inflammatory infiltrates in the stroma are often associated with invasive cervical cancers^14^ supported by the cytokine interleukin (IL)‐6 of HPV‐transformed keratinocytes^15^, ^16^, ^17^ favoring infiltration of T‐helper (Th)17 cells,^18^ an IL‐17‐expressing subgroup of Th cells.^19^ Strikingly, a hypoxic micromilieu favors the differentiation of Th17 cells controlled by hypoxia‐inducible factor 1 (HIF‐1).^20^ Th17 cells exhibit pro‐inflammatory as well as tumor‐promoting properties in different cancer types,^21^, ^22^ increase in the blood of cervical cancer patients^23^ and infiltrate cervical cancer tissues.^18^ Th17 cells activate the AKT signaling pathway in cervical cancer cells mediating reduced responsiveness of these cells toward chemotherapeutic drugs, irradiation, and combined treatment.^21^ Simultaneously, Th17 cells survive radio‐ or chemoradiotherapy in the blood of patients with cervical or vulva cancers.^21^, ^24^ Notably, the presence of Th17 cells in cervical cancer biopsies was associated with reduced recurrence‐free survival (RFS).^25^ Although hypoxia promotes Th17 differentiation and Th17 cells as well as hypoxia are both involved in AKT signaling pathway activation favoring tumor‐promoting mechanisms, a functional link between Th17 cells and hypoxia is not described so far.

In this study, we analyzed whether Th17 cells synergize with hypoxia during cervical cancer progression. We considered mechanisms and regulators induced by Th17‐hypoxia‐synergism in 2D cultures and 3D spheroids favoring cancer invasiveness as well as patients' materials to analyze Th17‐regulated factors as potential targets for therapy. We demonstrated that Th17 cells and hypoxia synergize in the expression of glycolytic enzymes and transporter under hypoxia in cervical cancer cells, functionally resulting in enhanced glucose uptake as well as proliferation, migration, and invasion. Our data provided evidence that Th17 cells induce the expression of IGF2BP2 in hypoxic cervical cancer cells and identified IGF2BP2 as the responsible mediator for enhanced proliferation, migration, and invasion using small molecule IGF2BP2 inhibitors of the benzamidobenzoic class. Notably, we found a heterogeneous IGF2BP2 expression in cervical cancer patients in situ correlating with infiltrating Th17 cells, and high IGF2BP2 expression was associated with lymph node metastases and relapse.

## 2 MATERIALS AND METHODS

### 2.1 Cell culture

HPV18‐positive cervical carcinoma cell lines SW756 (RRID:CVCL_1727), HeLa (RRID:CVCL0030) and HPV16‐positive SiHa cells (RRID:CVCL_0032) were received from ATCC (SW756, SiHa) or DSMZ (HeLa). All cell lines were authenticated using short tandem repeat (STR) profiling within the last 3 years and cultivated as described in Supporting Information Material and Methods. Hypoxia was induced by cobalt chloride (300 μM, Sigma‐Aldrich) or 1% O_2_ incubation (Binder, Tuttlingen, Germany). All experiments were performed with mycoplasma‐free cells.

### 2.2 Generation and cultivation of Th17 cells in vitro and ELISA

Th17 cells were generated as previously described.^18^ For conditioned media (CM), Th17 cells or naive CD4^+^ T cells were cultured at a density of 1 × 10^6^ cells/mL in RPMI‐1640 medium plus supplements (10% heat‐inactivated endotoxin‐tested FCS [Gibco] and 1 mmol/L sodium pyruvate). After 24 h, conditioned media were collected and analyzed for IL‐17A by DuoSet ELISA (R&D Systems, Minneapolis, MN, USA). Concentration of IL‐17A in the used CMTH17 was 227 pg/mL. Naive CD4^+^ T cells lacked IL‐17 expression. In stimulation experiments, CMs were used in a final dilution of 20% (vol/vol).

### 2.3 qRT‐PCR

RNA isolation, cDNA synthesis and real‐time PCR were performed as previously described.^26^ Via the PROBE FINDER software version 2.53 (Roche, Basel, Switzerland) PCR primers (Sigma‐Aldrich, Merck) were designed and listed in Supporting Information Material and Methods.

### 2.4 Protein expression analysis by Western blot analysis

The 4 × 10^5^ (SiHa), 5 × 10^5^ (SW756), and 3 × 10^5^ (HeLa) cells/6‐well culture dish were stimulated with medium, rhIL17 (100 ng/mL, Miltenyi Biotec), or CMTH17 and incubated under normoxic or hypoxic oxygen conditions. After 24 h, whole protein extracts were isolated as described in Ref.^26^. Exactly 15 μg of the whole protein extracts were separated by SDS gel electrophoresis and transferred to a nitrocellulose or PVDF membrane (Amersham). Rabbit anti‐hexokinase II, rabbit anti‐vimentin, mouse anti‐e‐cadherin, rabbit anti‐pAKTSer (all from CellSignaling, Massachusetts, USA), rabbit anti‐IGF2BP2, and mouse anti‐β‐actin antibody (both from Sigma‐Aldrich) were used. For the detection with the Molecular Imager ChemiDoc TM Touch Imaging System (Biorad), secondary antibodies (anti‐rabbit‐POX or anti‐mouse‐POX, Sigma‐Aldrich) as well as SuperSignal West Dura Extendet Duration Substrate (Thermo sctientific) were used. Protein expression was quantified with the Image Lab software (Bio‐Rad, Feldkirchen, Germany).

### 2.5 Microscopy

Assays with 2D cell cultures and 3D invasion of spheroids were documented with Leica DMI6000B or Leica DMI8 and LAS X software, as well as Leica DMIL with LASV4.8 software. Fluorescence microscopy was done using an Axio Imager KMAT microscope with ZEN3.1 software (Zeiss). Paraffin‐embedded cervical cancer biopsies were scanned with standardized settings using an Olympus BX51 microscope and VIS (Visiopharm Integrator System, Hørsholm, Denmark), Cell Sens Dimension, and Microsoft Image Composite Editor Program.

### 2.6 NBDG assay

The 5 × 10^4^ (SiHa), 6 × 10^4^ (SW756), and 4 × 10^4^ (HeLa) cells/24‐well culture dish were stimulated with rhIL17 (100 ng/mL), CMTH17 or medium as a control and incubated under normoxic and hypoxic oxygen conditions. After 24 h, medium was replaced by 2‐NBDG solution (10 mg/mL, 2‐(N‐[7‐nitrobenz‐2‐oxa‐1,3‐diazol‐4‐yl]amino)‐2‐deoxyglucose, Invitrogen). After 1 h, cells were washed with PBS, and fluorescence was documented and evaluated with ImageJ software.

### 2.7 Scratch assay

Monolayers of 5 × 10^5^ SiHa cells/6‐well were scratched, washed with PBS and stimulated with rhIL17 (100 ng/mL), CMTH17 or medium as a control. In hypoxia experiments, cells were treated with cobalt chloride daily (300 μM, Sigma‐Aldrich) or incubated in 1% O_2_. The scratches were analyzed at time points 0, 24, 48, and 72 h. The area of the scratch was quantified with ImageJ software (https://imagej.net/; RRID: SCR_003070). The area of the respective scratches at time point 0 was set at 100%. In scratch assays with IGF2BP2 inhibitors, 5 × 10^5^ SiHa cells/6‐well were pre‐incubated with IGF2BP2 inhibitors (50 μM) or DMSO as a control. These two compounds inhibiting IGF2BP2/RNA interactions (IGF2BP2 inhibitors#1, #2) were characterized in Ref. 27 and belong to the benzamidobenzoic acid class. After 1 h, cells were scratched and treated again with IGF2BP2 inhibitor (25 μM) or DMSO followed by simultaneous stimulation with rhIL17 (100 ng/mL), CMTH17 or medium for the time period of scratch documentation.

### 2.8 siRNA transfections

Cells were transfected as described in Supporting Information Material and Methods. After 24 h, cells were stimulated with rhIL17, CMTH17 or medium and incubated under normoxic or hypoxic oxygen conditions. In scratch assays, 4 × 10^5^ SiHa cells/6‐well were transfected. After 24 h, cells were scratched and stimulated as described above.

### 2.9 Transwell migration assay

For transwell migration assays, SiHa cells were stimulated with rhIL‐17 (100 ng/mL), CMTH17 or medium and cultured in normoxic or hypoxic oxygen conditions for 24 h. Cells were resuspended in DMEM without FKS and 3 × 10^4^ cells were seeded into 24‐well Transwell chambers (insert 6.5‐mm diameter, 8‐μm pore size; Corning Costar Corp., New York, NY, USA), loaded with DMEM containing 10% FKS. After 24 h, cells were fixed in 70% ethanol and stained with crystal violet. Cells were washed with aqua dest., and non‐migrated cells were removed from transwells using cotton swabs. The number of migrated cells was documented by six independent pictures per condition.

### 2.10 Spheroid formation and spheroid invasion assay

Spheroids were generated as previously described.^26^ On day 5, spheroids were treated with IGF2BP2 inhibitors (25 μM, inhibitor #1, #2) or DMSO as a control and stimulated with rhIL17, CMTH17 or medium as well as CM of naive CD4^+^ T cells as a control for 24 h. On day 6, spheroids were embedded in Matrigel Growth Factor Reduced (GFR) Basement Membrane Matrix (Corning Costar Corp, New York, USA). Invasion of spheroids was documented over 8 days. During invasion assays, on day 3 spheroids were treated again with inhibitors (25 μM) or DMSO as well as rhIL17, CMTH17, medium or CM of naive CD4^+^ T cells. The size of the spheroids was measured with ImageJ software. To determine the invasiveness of spheroids, the size of the spheroids on day 0 was subtracted from the size of the spheroids on day 8.

### 2.11 Protein expression analysis by immunofluorescence of 2D cells and 3D spheroids

For immunofluorescence (IF), 3 × 10^4^ cells were seeded on glass microscope slides with reaction wells (Marienfeld, Lauda‐Königshofen, Germany). Cells were stimulated with rhIL17 (100 ng/mL), CMTH17 or medium as a control and incubated under normoxic or hypoxic conditions. After 24 h, cells were fixed, permeabilized and stained with antibodies (rabbit anti‐CAIX, Abcam; rabbit anti‐SLC2A1, CellSignaling; rabbit anti‐HIF‐1A, Invitrogen) followed by anti‐rabbit Alexa Fluor 546‐conjugated secondary antibodies (Invitrogen) according to Ref. 18. For IF of spheroids, spheroids were generated in the presence of rhIL17, CMTH17 or medium as a control. After 11 days, spheroids were fixed in 4% paraformaldehyde and embedded in paraffin. Five‐μm‐thick sections were cut and stained with hematoxylin and eosin (HE) according to standard procedures or analyzed via IF as described in Supporting Information Material and Methods.

### 2.12 Tissue specimens, IHC and IF analysis

Formalin‐fixed paraffin‐embedded anonymized lesions of the cervix uteri from 45 patients were taken from the local pathology archive of the Saarland University Medical Center (Homburg, Germany). The tumors were staged according to FIGO classification by expert pathologists (Y.‐J. Kim or R.M. Bohle). Lesions were stained as described in Supporting Information Material and Methods. To evaluate the number of infiltrating Th17 cells, the numbers of CD4^+^ and IL‐17^+^ cells were counted and referred to numbers per mm^2^. IGF2BP2‐staining intensity was classified by using immunoreactive score (IRS, 0–2 = negative, 3–4 = weak, 6–8 = moderate, 9–12 = strong expression) (Table S1, ^28^).

### 2.13 Statistics

To analyze the statistical significance of the study's results, GRAPHPAD PRISM 8 (GRAPHPAD Software, Boston, MA, USA) RRID: SCR_000306 program was used. Nonparametric data of two groups were analyzed with a two‐tailed Mann–Whitney *U*‐test. Correlation between the IRS of IGF2BP2 and the number of Th17 cells was done using Spearman rank correlation. Best cutoffs to discriminate patients with increased Th17 frequencies and increased IGF2BP2 expression and with or without recurrent cervical cancers were identified by receiver operating characteristics (ROC) analysis and Youden's index calculation.

## 3 RESULTS

### 3.1 Th17 cells enhance hypoxia‐induced expression of CAIX, hexokinase II and SLC2A1 in cervical cancer cells

As hypoxic cervical cancer cells showed enhanced AKT activation^10^ and Th17 cells also contribute to AKT signaling pathway induction in cervical cancer cells favoring therapy resistance and cancer progression,^21^ we were interested whether Th17 cells synergize with hypoxia‐related mechanisms affecting cancer progression. In line with previous results,^10^ cultivation of cervical cancer cells under hypoxic conditions resulted in induction of AKT phosphorylation at residue S473 (Figure S1A,B). Interestingly, stimulation of hypoxic cells with rhIL‐17 or conditioned media of Th17 cells (CMTH17) further increased pAKTSerin expression (Figure S1A,B) indicating a synergistic interaction.

**FIGURE 1:**
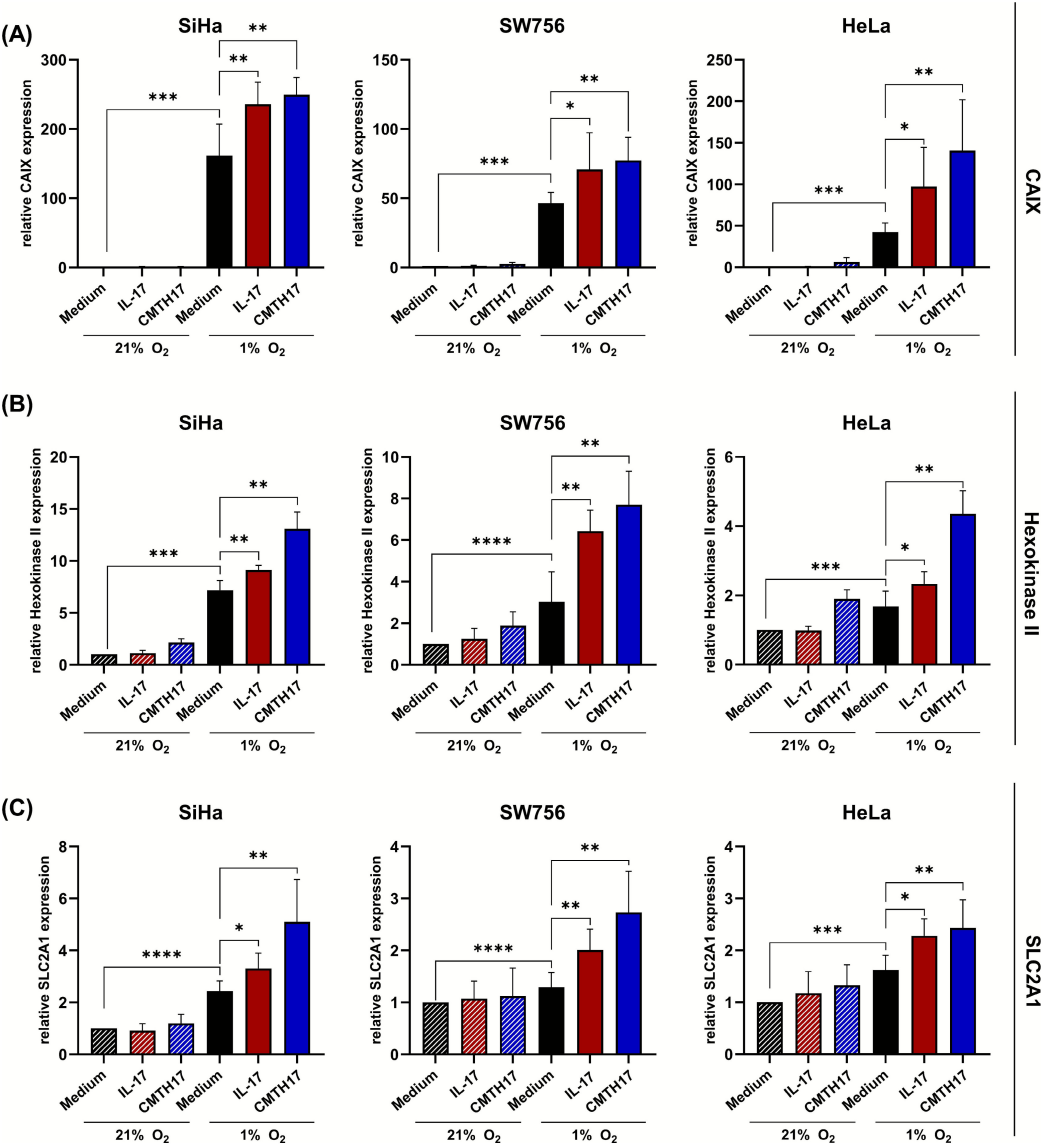
Th17 cells induce CAIX, hexokinase II and SLC2A1 mRNA expression in an oxygen‐dependent manner. SiHa (left), SW756 (middle) and Hela (right) were stimulated with medium (black bars), rhIL‐17 (100 ng/mL, red bars) and conditioned media of in vitro generated Th17 cells (CMTH17, 20%, blue bars) and incubated under normoxic (21% O_2_, striped bars) or hypoxic oxygen conditions (1% O_2_). After 24 h, the cells were analyzed for (A) CAIX, (B) hexokinase II and (C) SLC2A1 expression by qRT‐PCR analysis and normalized to RPL13A housekeeping gene expression. The quotient of the gen of interest/RPL13A of medium‐ stimulated cells incubated by 21% oxygen was set at 1. Shown are the results (mean + SD) from six independent stimulations. Asterisks (*p < .05, **p < .01, ***p < .001, ****p < .0001) represent statistical significances. The *p*‐value according to the nonparametric Mann–Whitney *U*‐test.

To adapt to hypoxic environment and attended metabolic changes in glucose consumption, hypoxic cells induce the expression of glycolytic enzymes and hypoxic markers, including hexokinase II, glucose transporter 1 and carbonic anhydrase IX (CAIX).^29^, ^30^ We validated induction of the hypoxic marker HIF‐1α (Figure S2A–C) as well as CAIX, hexokinase II and SLC2A1 expression by reduced oxygen concentration in cervical cancer cells on mRNA levels (Figure 1A–C; black bars; up to 162‐fold increased expression) in comparison to control cells cultured by 21% O_2_ (stripped black bars). Stimulation of normoxic cells with rhIL17 (stripped red bars) or CMTH17 (stripped blue bars) resulted in 1.2‐ to 6.5‐fold increased CAIX, hexokinase II and SLC2A1 expression. Interestingly, there was a further enhanced expression of hypoxic markers in cervical cancer cells under 1% O_2_ after stimulation with rhIL17 (red bars, 1.3‐ to 2.1‐fold increase) and to a higher extent after CMTH17 stimulation (blue bars, 1.5‐ to 3.3‐fold increase). In conclusion, our data demonstrate that Th17 cells support mRNA expression of glycolytic enzymes and transporters under normoxic conditions; however, this effect is reinforced in hypoxic cervical cancer cells.

**FIGURE 2:**
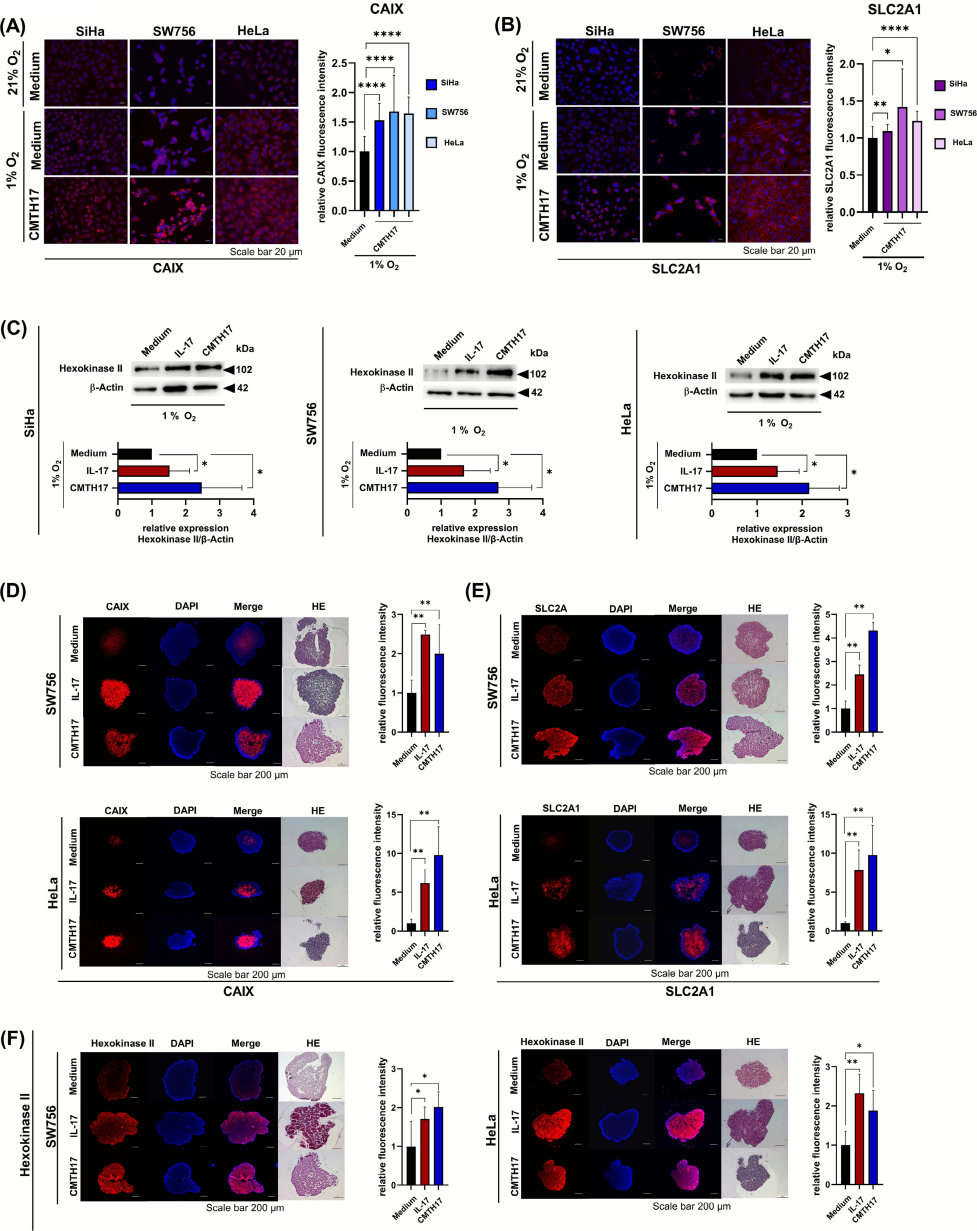
Th17‐induced expression of CAIX, SLC2A1 and hexokinase II on protein level in 2D cultures and 3D spheroids. (A, B) 2D monolayers of SiHa, SW756 and HeLa were stimulated with medium or CMTH17 and incubated under hypoxic conditions. Twenty‐four hours later CAIX (A, blue bars) and SLC2A1 (B, purple bars) expression were investigated by IF. Bars represent quantification of relative fluorescence/cell of 20 independent pictures (scale bar: 20 μm) From *n* = 2 independent experiments. The values of medium‐stimulated cells by 21% oxygen was set at 1%. (C) SiHa (left), SW756 (middle) and HeLa cells (right) were stimulated with 100 ng/mL rhIL‐17 (red bars) or 20% CMTH17 (blue bars) for 24 h by hypoxic conditions. Whole cell extracts were analyzed for hexokinase II expression by Western blot analysis. The relative hexokinase II expression (hexokinase/β‐Actin) in cells incubated by 1% oxygen was set at 1. β‐Actin was used as a loading control. Shown are the results (mean + SD) from four independent stimulations. (D–F) 3D spheroids of SW756 and HeLa were generated over 11 days in the presence of medium, rhIL‐17 (red bars) or CMTH17 (blue bars). 5 μm sections of fixed paraffin‐embedded spheroids were validated by HE stainings and analyzed for (D) CAIX, (E) SLC2A1 and (F) hexokinase II expression by IF (scale bar: 200 μm). Sections from CMTH17‐stimulated SW756 spheroids were used for CAIX (D) and hexokinase II (F) stainings, sections from medium‐stimulated HeLa spheroids were used for CAIX (D) and SLC2A1 (E) stainings, sections from IL‐17‐stimulated HeLa spheroids were used for SLC2A1 (E) and hexokinase II (F) stainings and sections from CMTH17‐stimulated HeLa spheroids were used for SLC2A1 (E) and hexokinase II (F) stainings. Bars represent quantification of relative fluorescence/spheroid of *n* = 6 independent spheroids, respectively (mean + SD). Asterisks (*p < .05, **p < .01, ****p < .0001) represent statistical significances. The *p*‐value according to the nonparametric Mann–Whitney *U*‐test.

We validated these findings on protein levels by IF and Western blot analysis. In IF, stimulation of three different cervical cancer cell lines with CMTH17 enhanced CAIX expression up to 68% (Figure 2A, blue bars) and SLC2A1 expression up to 42% (Figure 2B, purple bars). This Th17‐induced effect was also found for hexokinase II protein expression in three different cell lines (Figure 2C, up to 2.7‐fold increase) by Western blot analysis.

Next, we generated spheroids of SW756 and HeLa cells (Figure 2D–F) and analyzed hypoxic marker expression in paraffin‐embedded slices by IF. Our results demonstrated hypoxic marker expression (CAIX, SLC2A1, hexokinase II) predominantly in the inner part of unstimulated cervical cancer spheroids (upper panels, respectively). Stimulation of spheroids with rhIL17 and CMTH17 resulted in an increase in hypoxic marker expression (1.7‐ to 9.8‐fold) extending to outer regions of the spheroids (Figure 2D,E). In summary, our results showed that Th17 cells increase mRNA and protein expression of hypoxia‐related glycolytic enzymes and transporters, especially under hypoxic oxygen conditions in 2D as well as 3D cultured cervical cancer cells.

### 3.2 Th17 cells enhance glucose uptake as well as proliferation and migration of cervical cancer cells under hypoxic conditions

**FIGURE 3:**
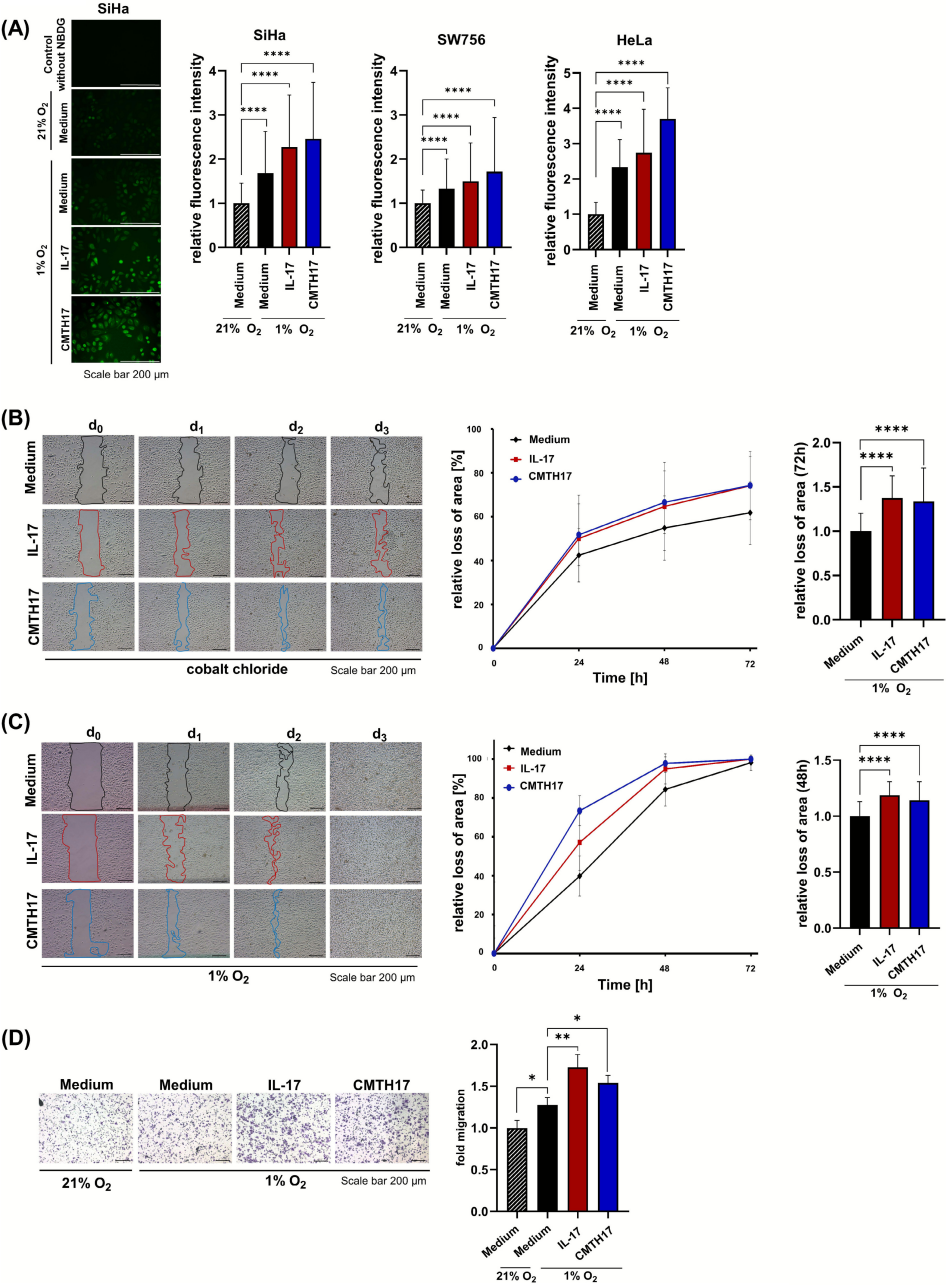
Th17 cells mediate enhanced glucose uptake as well as proliferation and migration of cervical cancer cells under hypoxic conditions. (A) HeLa, SiHa and SW756 cells were stimulated with rhIL‐17 (100 ng) or CMTH17 and cultured in normoxic (21% oxygen) or hypoxic (1% oxygen) conditions. After 24 h, the cells were incubated with the fluorescent glucose analogon NBDG (10 mg/mL diluted in PBS) for 1 h. Glucose uptake was investigated by IF. Bars represent quantification of relative fluorescence/cell of 150 cells in total from five independent pictures (scale bar: 200 μm) of independent stimulation experiments, respectively. The values of medium‐stimulated cells incubated by 21% oxygen was set at 1. (B, C) Monolayers of SiHa cells stimulated with medium (black lines), rhIL‐17 (red lines), CMTH17 (blue lines) were scratched and (B) stimulated with 300 μM cobalt chloride every 24 h to create and retain hypoxic conditions or (C) incubated by hypoxic conditions (1% O_2_). Pictures of the scratches were taken after 0, 24, 48, and 72 h (scale bar: 200 μm). The relative loss of area was calculated for (B) 3 days or (C) 2 days, respectively, indicated by lines (left picture), in relation to time point 0 h (middle, line graphics). The relative loss of the area after 72 or 48 h of the cells stimulated with medium was set at 1 (right, bar chart). The line graphics show one representative experiment performed in doubles (mean ± SD). Bars represent data (mean + SD) of *n* = 3 independent experiments performed in doubles. (D) SiHa cells were stimulated with medium (black bars), rhIL‐17 (red bars) or CMTH17 (blue bars) and cultured in normoxic (21% oxygen) or hypoxic (1% oxygen) conditions. After 24 h, cells were used in transwell migration assays. Transmigrated cells were calculated after 24 h. Representative pictures (left panel; scale bar: 100 μm); Quantification of *n* = 3 experiments with six independent pictures, respectively (mean ± SD), lower panel. The number of medium stimulated normoxic cells was set at 1. Asterisks (*p < .05, **p < .01, ****p < .0001) represent statistical significances. The *p*‐value according to the nonparametric Mann–Whitney *U*‐test.

Results of mRNA and protein expression levels prompted us to analyze functional consequences of synergistic mechanisms between Th17 cells and hypoxia. Due to the fact that Th17 cells enhance SLC2A1 expression in cervical cancer cells (Figures 1 and 2) and the ability of cancer cells to metabolize glucose in the absence of oxygen because of HIF‐1α‐mediated adaption to hypoxic conditions,^31^ we first analyzed the ability of Th17‐hypoxia‐instructed cervical cancer cells for glucose uptake by IF using the fluorescent glucose analogon NBDG (Figure 3A). Cultivation of cervical cancer cells in 1% O_2_ conditions (black bars) mediated enhanced NBDG uptake (1.3‐ to 2.3‐fold increase) in comparison to normoxic cervical cancer cells (stripped black bars). Additionally, stimulation of three different cervical cancer cell lines under hypoxic conditions with rhIL17 (red bars: 1.5‐ to 2.7‐fold) or CMTH17 (blue bars: 1.7‐ to 3.7‐fold) further increased NBDG uptake in comparison to unstimulated cells (Figure 3A).

Our results and the fact that glucose is an energy source favoring proliferation and migration of bladder cancer cells^32^ prompted us to analyze whether Th17 cells and hypoxia affect proliferation and migration of cervical cancer cells. This idea was strengthened by the fact that Th17‐hypoxia‐stimulation favors reduced e‐cadherin but enhances vimentin expression in cervical cancer cells (Figure S3A,B). In a scratch assay, measuring both proliferation and migration, we used cobalt chloride in a first experimental setup (Figure 3B) to induce hypoxic conditions validated by enhanced HIF‐1α expression (Figure S4A,B). After stimulation with rhIL17 (red lines and bars) or CMTH17 (blue lines and bars), the introduced gap was reduced significantly faster resulting in a significant relative loss of area after 72 h by 37% or 34%, respectively, in comparison to unstimulated hypoxic cells. We validated these results in a second experimental setup culturing the cells by 1% oxygen (Figure 3C). Again, the introduced gap was closed significantly faster after stimulation of cervical cancer cells with rhIL17 (red lines, bars) and CMTH17 (blue lines, bars) resulting in 19% or 14% enhanced gap closure after 48 h. The enhanced potential for migration of Th17‐hypoxia‐stimulated cells went along with a stabilized reduced e‐cadherin and enhanced vimentin expression in cervical cancer cells after 3 days of stimulation supporting our findings (Figure S3C). In transwell migration assays, cultivation of cervical cancer cells in 1% oxygen resulted in 28% enhanced migration in comparison to normoxic control cells (Figure 3D). Stimulation of cervical cancer cells with rhIL17 (red bar) or CMTH17 (blue bar) further increased migration of hypoxic cervical cancer cells (up to 40% increase). Thus, our results demonstrated Th17‐mediated increased glucose uptake as well as proliferation and migration of hypoxic cervical cancer cells.

### 3.3 Th17 cells increase IGF2BP2 expression in hypoxic cervical cancer cells

**FIGURE 4:**
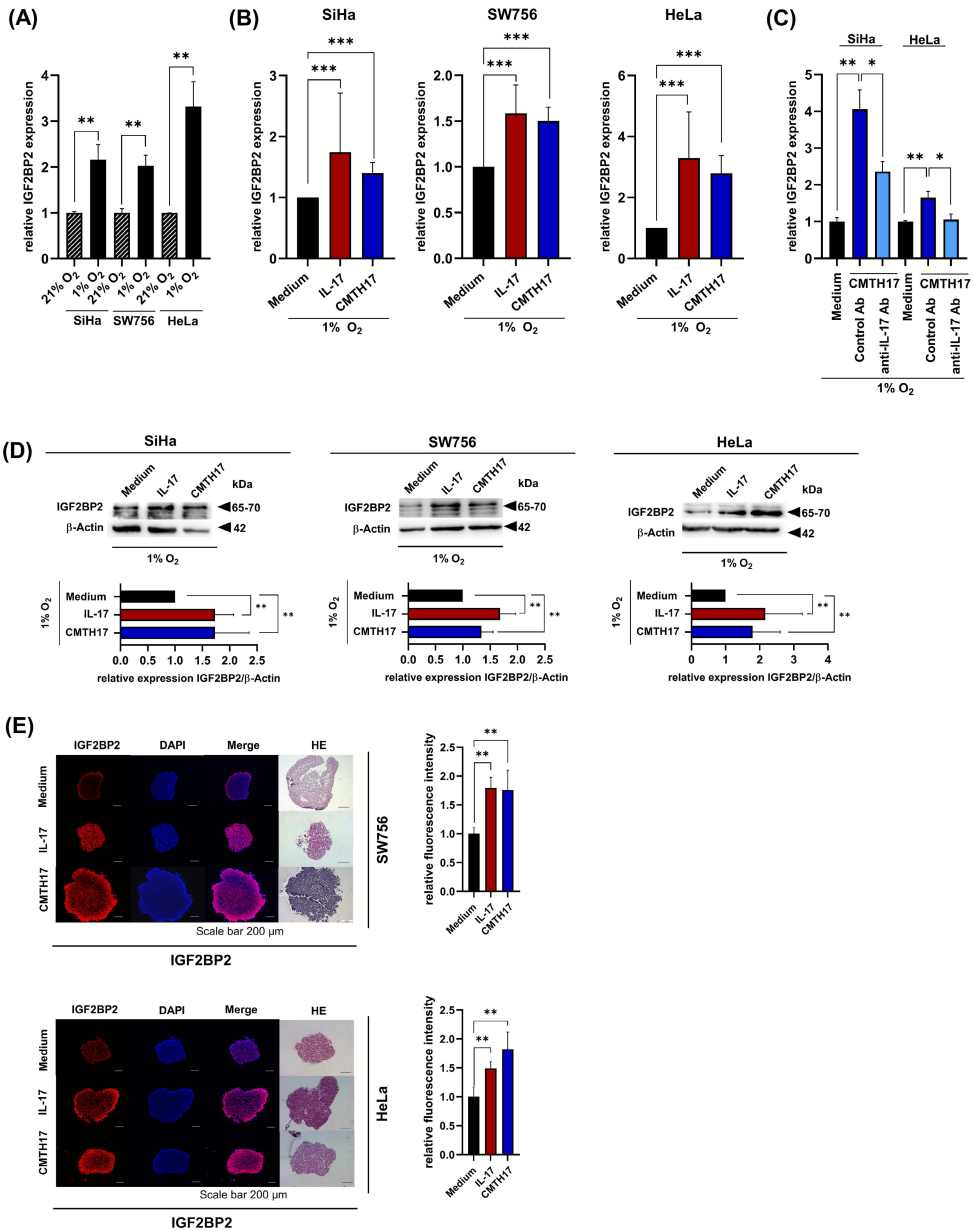
Th17 cells increase IGF2BP2 expression on mRNA and protein expression levels in hypoxic cervical cancer cells. (A) SiHa, SW756 and HeLa cells were incubated by normoxic (21%) or hypoxic oxygen conditions (1% O_2_). After 24 h, the cells were analyzed for IGF2BP2 expression by qRT‐PCR analysis and normalized to RPL13A housekeeping gene expression. The quotient of IFG2BP2/RPL13A of normoxic cells was set at 1. Shown are the results (mean + SD) from four independent experiments. (B, C) SiHa (left), SW756 (middle) and Hela (right) were stimulated with medium (black bars), rhIL‐17 (100 ng/mL, red bars) and conditioned media of in vitro generated Th17 cells (CMTH17, 20%, blue bars) and incubated by hypoxic oxygen conditions (1% O_2_). (B) After 24 h, the cells were analyzed for IGF2BP2 expression by qRT‐PCR analysis and normalized to RPL13A housekeeping gene expression. The quotient of IFG2BP2/RPL13A of medium stimulated cells was set at 1. Shown are the results (mean + SD) from six independent stimulations. (C) In neutralization experiments, CM were prestimulated with neutralizing anti‐IL‐17 or respective isotype control antibodies for 2 h (light blue bars) and IGF2BP2 expression was analyzed in relation to RPL13A. (D) After 24 h, whole cell extracts were analyzed for IGF2BP2 expression by Western blot analysis. β‐Actin was used as a loading control. The relative IGF2BP2 expression (IGF2BP2/ β‐Actin) of medium stimulated cells was set at 1. Bars represent results (mean + SD) from four independent stimulations. (E) 3D spheroids of HeLa and SW756 were generated over 11 days in the presence of medium, rhIL‐17 (red bars) or CM of Th17 cells (blue bars). The 5‐μm sections of fixed paraffin‐embedded spheroids were validated by HE stainings and analyzed for IGF2BP2 expression by IF (scale bar: 200 μm). Sections from medium‐stimulated SW756 spheroids from Figure 2F were used for IGF2BP2 stainings, sections from medium‐stimulated HeLa spheroids from Figure 2F were used for IGF2BP2 stainings and sections from IL‐17‐stimulated HeLa spheroids from Figure 2E,F were used for IGF2BP2 stainings. Bars represent quantification of relative fluorescence/spheroid of *n* = 6 independent spheroids, respectively (mean + SD). Asterisks (*p < .05, **p < .01, ***p < .001) represent statistical significances. The *p*‐value according to the nonparametric Mann–Whitney *U*‐test.

Next, we were interested in the mechanism underlying Th17‐induced enhanced proliferation and migration of hypoxic cervical cancer cells. A mRNA expression analysis, investigating IL‐17‐ or CMTH17‐mediated mRNA expression changes (Agilent SurePrint G3 Human GE v3 8 × 60K microarray),^25^ revealed Th17‐mediated up‐regulation of IFG2BP2/IMP2 in normoxic cervical cancer cells (16%–23% increase, data not shown). The insulin‐like growth factor 2 mRNA binding protein 2 (IGF2BP2), a RNA‐binding protein, is overexpressed in several tumor entities, dedicated to influence glycolysis through stabilizing glycolytic enzymes, like SLC2A1 affecting glycolysis rate,^33^ and described as regulator of EMT‐related factors.^34^ Thus, IGF2BP2 seems to be an interesting candidate. Our results demonstrated up‐regulation of IGF2BP2 mRNA expression in hypoxic cervical cancer cells in comparison to normoxic cells (Figure 4A; 2‐ to 3.3‐fold increase) that was further enhanced after stimulation with rhIL17 (Figure 4B, red bars: 1.6‐ to 3.3‐fold increase) or CMTH17 (blue bars: 1.4‐ to 2.8‐fold increase). Neutralization of IL‐17 in the CM of Th17 cells significantly reduced IGF2BP2 expression (Figure 4C; 41%–92% reduction) in 45.5%–92% reduction clearly demonstrating that induction of IGF2BP2 was dependent on Th17‐derived IL‐17. IL‐17‐ or CMTH17‐induced IGF2BP2 expression was validated on protein levels by Western blot analysis (Figure 4D; red bars: 1.7‐ to 2.2‐fold increase, blue bars: 1.3‐ to 1.8‐fold increase). Furthermore, IF of slides from 3D spheroids revealed rhIL17‐ (red bars: 1.5‐ to 1.8‐fold) and CMTH17‐mediated (blue bars: 1.8‐fold) increase in IGF2BP2 protein expression (Figure 4E). Interestingly, Th17 cells induced IGF2BP2 protein expression in Th17‐cervical cancer cells hetero‐spheroids, too (Figure S4C; 37% increase), however, to a minor degree than induction by rhIL‐17 or CMTH17 cells. In summary, our findings showed that Th17‐induced IGF2BP2 mRNA and protein expression in hypoxic cervical cancer cells was stronger by soluble factors produced by Th17 cells than direct co‐cultures and mediated via IL‐17.

### 3.4 Enhanced migration and invasion of hypoxic cervical cancer cells induced by Th17 cells is dependent on IGF2BP2

**FIGURE 5:**
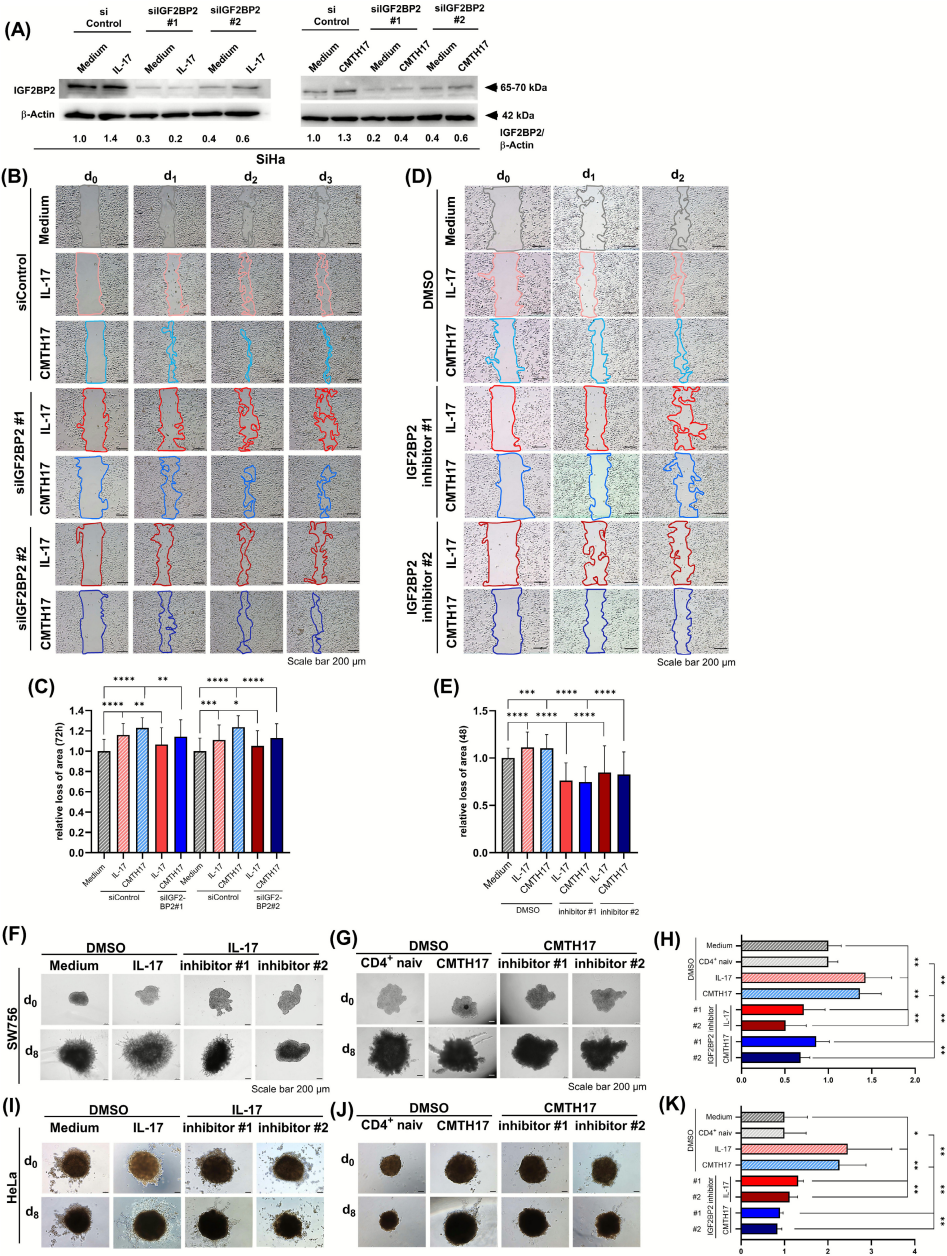
Th17‐hypoxia‐induced enhanced migration and invasion is dependent on IGF2BP2. (A) SiHa cells were transfected with IGF2BP2‐specific siRNA (siIGF2BP2 #1, #2) or siControl and stimulated with 100 ng/mL rhIL‐17 or CMTH17 for 24 h under hypoxic conditions. Whole cell extracts were analyzed for IGF2BP2 expression by Western blot analysis. β‐Actin was used as a loading control. The relative IGF2BP2 expression (IGF2BP2/β‐Actin) of medium stimulated cells was set at 1. (B, C) SiIGF2BP2 (#1, #2) transfected SiHa cells were scratched and stimulated with medium, rhIL‐17 or CMTH17 24 h post‐transfection. (B) Pictures of the scratches were taken after 0, 24, 48, and 72 h (scale bar: 200 μm). The relative loss of area was calculated for 3 days, indicated by lines, in relation to time point 0 h. The relative loss of the area after 72 h of the cells stimulated with medium was set at 1. (C) Bars represent data (mean + SD) of *n* = 3 independent experiments performed in doubles. (D, E) SiHa cells were stimulated with IGF2BP2 inhibitors (IGF2BP2 inihibitors#1, #2, 50 μM) for 1 h. Cells were scratched followed by stimulation with medium, rhIL‐17 or CMTH17 in the presence of IGF2BP2 inhibitors (IGF2BP2 inihibitors#1, #2, 25 μM for 72 h). Pictures of the scratches were taken after 0, 24, and 48 h (scale bar: 200 μm). The relative loss of area was calculated for 2 days, indicated by lines, in relation to time point 0 h. The relative loss of the area after 48 h of the cells stimulated with medium was set at 1. (E) Bars represent data (mean + SD) of *n* = 3 independent experiments performed in doubles. (F–H) Spheroids of SW756 cells and (I–K) spheroids of HeLa cells were generated in the absence or presence of rhIL‐17, CMTH17 cells, medium or CM of naive CD4^+^ cells. On day 5, spheroids were incubated with inhibitors (#1/#2, 25 μM) or DMSO as a control for 24 h. On day 6, spheroids were embedded into Matrigel. Pictures were taken for 8 days and spheroid invasion was calculated. Shown are the pictures of one representative experiment which depicts the invasiveness of the spheroids over the time (F–J, scale bar: 200 μm). The relative invasiveness after 8 days was determined in relation to medium or CM of naive CD4^+^ T cells, respectively and DMSO stimulated cells which was set at 1 (H, K, gray stripped bars). Due to the invasiveness of spheroids from HeLa cells on day 8, pictures were merged with the Microsoft Image Composite Editor program. Shown are the results (mean + SD) from six independent spheroids. Asterisks (*p < .05, **p < .01, ***p < .001, ****p < .0001, n.s. = not significant) represent statistical significances. The *p*‐value according to the nonparametric Mann–Whitney *U*‐test.

To analyze the relevance of IGF2BP2 for migration of Th17‐hypoxia‐instructed cervical cancer cells, we knocked down IGF2BP2 with two specific siRNAs, resulting in reduced IGF2BP2 expression after rhIL17 or CMTH17 stimulation on mRNA up to 78.1% reduction (Figure S5A) and protein levels (ranged from 74.9% to 90.1% after rhIL17 and 57.4% to 86.9% after CMTH17 stimulation; Figures 5A and S5B,C) in two cervical cancer cell lines. In scratch assays with SiHa cells cultured in the presence of 1% O_2_, stimulation of siControl transfected cells with rhIL17 (red stripped bars) or CMTH17 (blue stripped bars) resulted in faster closure of the gap (Figure 5B,C). In contrast, transfection of IGF2BP2 specific siRNAs significantly reduced proliferation and migration of rhIL17‐ (red and dark red bars) and CMTH17‐instructed (blue and dark blue bars) hypoxic cervical cancer cells and decreased rhIL17‐ and CMTH17‐mediated loss of area by 50%–55% or 39%–55%, respectively (Figure 5C).

In addition to siRNAs, we used recently described small‐molecule inhibitors of IGF2BP2 from the benzamidobenzoic acid class to block interactions of IGF2BP2 with its RNA targets.^27^ The compounds did not affect endogenous IGF2BP2 expression in cervical cancer cells (Figure S5D) but reduced the abundance of the IGF2BP2 target gene *SLC2A1* and hexokinase II (Figure S5E) in the same way as after siRNA‐mediated knock‐down of IGF2BP2 (Figure S5F,G). In scratch assays, two different IGF2BP2 inhibitors significantly reduced proliferation and migration of hypoxic rhIL17‐ or CMTH17‐instructed cervical cancer cells (35.5%–30.1% reduction, red and blue bars) in comparison to DMSO‐treated control cells (stripped red and blue bars, Figure 5D,E). Furthermore, we applied these compounds to 3D spheroids from cervical cancer cells exhibiting IGF2BP2 expression (Figure 4C). IGF2BP2 inhibitors completely abolished rhIL17‐ or CMTH17‐induced invasion of 3D spheroids after 8 days (Figure 5F–K). In summary, our data clearly proved that Th17‐induced enhanced migration and invasion of hypoxic cervical cancer cells is dependent on IGF2BP2 expression and function.

### 3.5 Th17 cells correlate with IGF2BP2 expression in cervical cancer biopsies associated with lymph node metastases and recurrent cervical cancers

**FIGURE 6:**
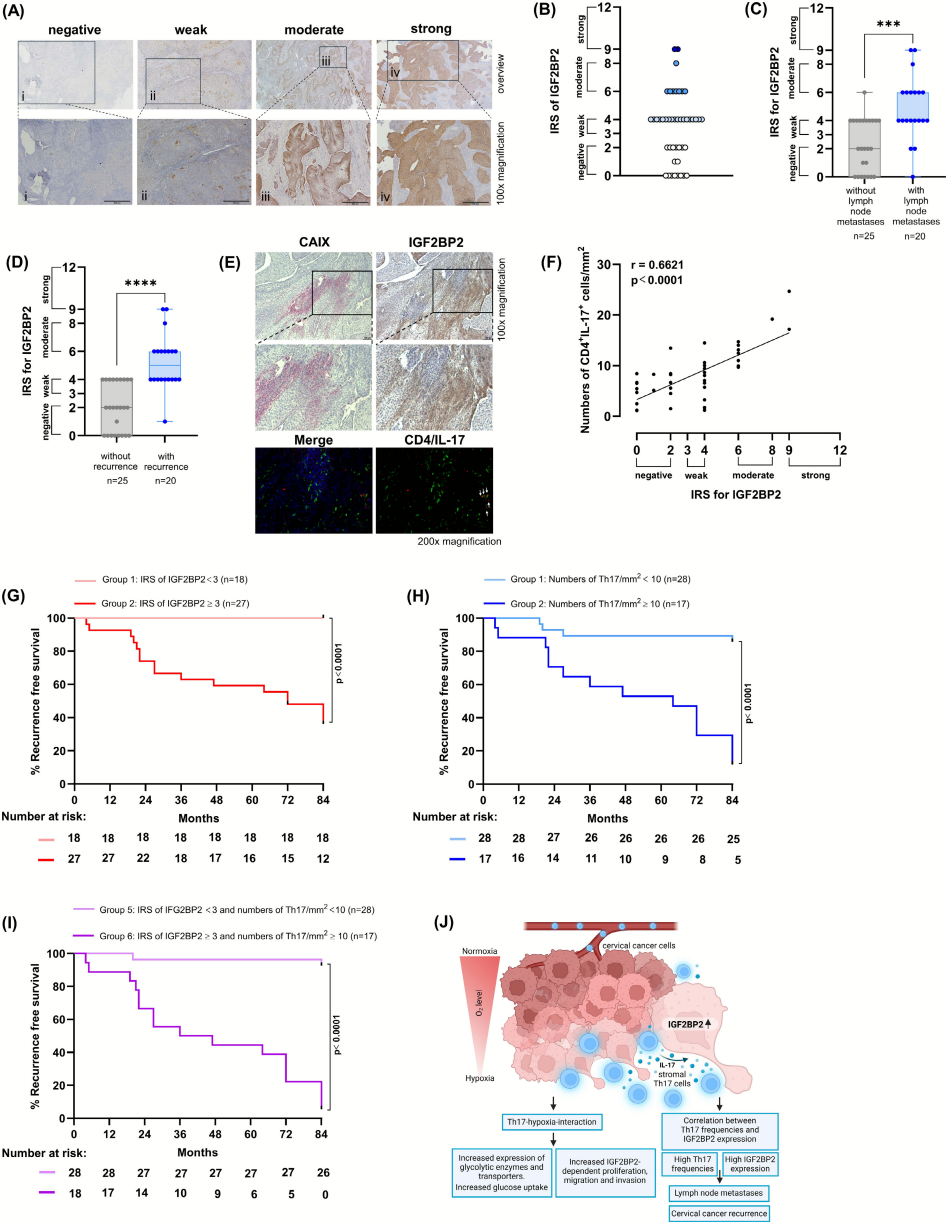
Th17 cells correlate with IGF2BP2 expression in cervical cancer biopsies in situ and are associated with lymph node metastases and cervical cancer recurrence. (A) Sections of human squamous cell carcinomas (SCCs) of 45 cervical cancer patients were stained for IGF2BP2 expression by IHC ((i) negative, (ii) weak, (iii) moderate, (iv) strong expression) (scale bar 500 μm). (B) IRS of IGF2BP2 within 45 biopsies. (C) IRS of IGF2BP2 in tumor tissues of patients with (blue circles) and without (gray diamonds) lymph node metastases or (D) with and without relapse. (E) Sections of human SCCs were stained for CAIX (pink) and IFG2BP2 (brown) or co‐stained for CD4 (green) and IL‐17 (A). Arrows indicate Th17 cells, scale bar 100 μm. (F) Correlation of numbers of Th17 cells/mm^2^ with IRS of IGF2BP2. The *p*‐value according to the nonparametric Mann–Whitney *U*‐test (C, D) or Spearman rank correlation with linear regression (F). Asterisks (***p < .001; ****p < .0001) represent statistical significances. (G) Recurrence‐free survival of 45 patients was determined for a cohort with IGF2BP2 expression (weak, moderate and strong, IRS ≥3; *n* = 27; red line) and negative IGF2BP2 expression (IRS <3; *n* = 18; light red line). Median recurrence‐free survival was 72 months for the cohort with IGF2BP2 expression. Comparison of survival analysis was performed using log‐rank (Mantel–Cox) test; chi‐square: 17.19, ***p < .0001. (H) Recurrence‐free survival of 45 patients was determined for a cohort with Th17 numbers/mm^2^ < 10 (*n* = 28; light blue line) and Th17 numbers/mm^2^ ≥ 10 (*n* = 17; blue line). Median recurrence‐free survival was 64 months for the cohort with Th17 numbers/mm^2^ ≥ 10. Comparison of survival analysis was performed using log‐rank (Mantel–Cox) test; chi‐square: 25.31, ***p < .0001. (I) Recurrence‐free survival of 45 patients was determined for a cohort with negative IGF2BP2 expression (IRS <3) and Th17 numbers/mm^2^ < 10 (*n* = 28; light purple line) and for a cohort with IGF2BP2 expression (weak, moderate and strong, IRS ≥3) and Th17 numbers/mm^2^ ≥ 10 (*n* = 17; purple line). Median recurrence‐free survival was 41.5 months for the cohort with IGF2BP2 expression and Th17 numbers/mm^2^ ≥ 10. Comparison of survival analysis was performed using log‐rank (Mantel–Cox) test; Chi‐square: 40.19, ***p < .0001. (J) Schematic presentation of the role of Th17‐hypoxia‐interactions in cervical cancer progression: Th17‐hypoxia‐induced IGF2BP2 expression favors cervical cancer invasiveness and increased frequencies of Th17 cells and IGF2BP2 expression related to cervical cancer recurrence (scheme was generated with the Biorender software, Toronto, Ontario, Canada).

To validate our in vitro findings, we analyzed IGF2BP2 expression by IHC in 45 clinical biopsies in situ (Figure 6A). Applying the immunoreactive score (IRS), we found a heterogenous IGF2BP2 expression in situ reaching from negative to strong (Figure 6B). Thirty‐eight percent of the clinical biopsies were negative (Figure 6A,i), whereas 62% were positive with weak (*n* = 18, Figure 6A,ii), moderate (*n* = 8, Figure 6A,iii) or strong (*n* = 2, Figure 6A,iv) IGF2BP2 expression. Interestingly, patients with lymph node metastases showed significantly higher IGF2BP2 expression in their tumor tissues in comparison to patients without lymph node metastases (Figure 6C). Moreover, a retrospective analysis demonstrated that in patients developing recurrence of cervical cancers IGF2BP2 expression is significantly more frequent in their tumor tissues than in patients without relapse (Figure 6D). IGF2BP2 expression was found in CAIX positive regions of tumors as well as tumor borders contacted with Th17‐infiltrated stroma (Figure 6E). Analyzing the relation between the presence of Th17 cells and IGF2BP2 expression, our patients' biopsies showed a significant correlation between the numbers of tumor‐infiltrating Th17 cells per mm^2^ and IRS of IGF2BP2 (Figure 6F; *r* = .6621; *p* < .0001). Thus, our results indicated a correlation between Th17 cells and IGF2BP2 expression in cervical cancer biopsies associated with lymph node metastasis and relapse.

To evaluate whether the presence of Th17 cells and IGF2BP2 expression is linked to the course of disease, ROC analyses were performed to identify a cut‐off value concerning IGF2BP2 expression and Th17 numbers, which discriminates between patients with and without recurrent cervical cancers. Best discrimination was obtained for a cut‐off value of an IRS score ≥3 (weak, moderate or strong IGF2BP2 expression; 95% sensitivity and 66% specificity; Figure S6A) and numbers of Th17/mm^2^ ≥ 10 (71.4% sensitivity and 91.3% specificity; Figure S6B). The area under the ROC curve (AUC) value was 0.8860 and 0.8602, respectively.

Applying the defined cut‐off values for 45 patients, in the group of patients with negative IGF2BP2 expression (light red line) RFS rate was 100% after 7 years, while for patients with weak, moderate or strong IGF2BP2 expression (red line) 3.5‐ and 7‐year RFS rate was 62.9% and 36.1%, respectively (Figure 6G). For patients with numbers of Th17/mm^2^ < 10 (light blue line) 7‐year RFS rate was 85.7% and for patients with numbers of Th17/mm^2^ ≥ 10 (blue line) 3.5‐ and 7‐year RFS rate was 58.8% and 11.8%, respectively (Figure 6H). Interestingly, after combination of both factors, IGF2BP2 expression and Th17 numbers, in the group of patients with negative IGF2BP2 expression and Th17/mm^2^ < 10 (light purple line) 6‐year RFS rate was 92.6%. Unlike, in the group of patients with weak, moderate and strong IGF2BP2 expression and Th17/mm^2^ ≥ 10 (purple line), 3.5‐ and 7‐year RFS rate was 50% and 5.6%, respectively (Figure 6I). This stratification of patients regarding IGF2BP2 expression (chi‐square: 17.19, *p* < .0001) or combined with Th17/mm^2^ was more efficient (chi‐square: 40.19, *p* < .0001) than applying discrimination based on another risk factor, the lymph node status (chi‐square: 8.323, *p* = .0039), resulting in 7‐year RFS from patients with lymph node metastases of 39.7% and 7‐year RFS from patients without lymph node metastases of 82.6% (Figure S6C). In conclusion, our data showed a clear association between Th17 levels as well as enhanced IGF2BP2 expression and cervical cancer recurrence.

## 4 DISCUSSION

Cervical cancer progression is affected by the tumor environment including areas with low oxygen concentration, termed hypoxic regions, as well as regions with chronic inflammation. Th17 cells represent a part of the inflammatory immune milieu of cervical cancers^18^, ^35^ and their differentiation is favored by low oxygen concentration.^20^ As hypoxic regions as well as Th17 cells are both associated with the activation of the pro‐tumorigenic AKT signaling pathway,^10^, ^21^ therapy resistance in cervical cancers^21^, ^36^ and poor prognosis of patients,^8^, ^17^ in this study we investigated interactions of both as a basis for new therapeutic approaches in cervical cancers. We could show that Th17 cells enhance the expression of hypoxia‐induced glycolysis‐related enzymes in hypoxic cervical cancer cells and promote their glucose uptake, proliferation and migration. We identified the RNA binding protein IGF2BP2 as a new regulator of Th17‐hypoxia‐mediated enhanced proliferation, migration and invasive behavior of cervical cancer cells. Notably, in situ analyses revealed a correlation between numbers of Th17 cells and IGF2BP2 expression that was associated with cervical cancer relapse and reduced recurrence‐free survival of patients. Figure 6J summarizes our current idea of a novel Th17‐hypoxia‐IGF2BP2‐mediated mechanism promoting cervical cancer progression.

The constitution of the individual cervical cancer microenvironment combined with chronic inflammation affects cervical cancer progression and correlates with clinical outcome.^14^ Our previous studies identified mechanisms how tumor‐fibroblast interactions favor the infiltration^18^ and expansion of Th17 cells in cervical cancer.^17^ A controversial role is supposed for Th17 cells in different cancers, with Th17‐mediated antitumor effects based on recruitment of immune cells into tumors or stimulating effector CD8^+^ T cells,^37^ as well as Th17‐based tumor‐promoting responses favoring proliferation, invasion, metastasis, and angiogenesis.^38^, ^39^ Our previous in situ analysis linked the presence of Th17 cells with cancer progression,^21^ lymph node metastases and cervical cancer recurrence.^17^ Furthermore, Th17 cells activate the AKT signaling pathway in cervical cancer cells reducing their responsiveness toward chemoradiotherapy.^21^ This pathway is also induced by hypoxia in cervical cancer cells^10^ and linked with poor prognosis of patients.^8^ In this study, we demonstrated first results of a Th17‐hypoxia‐synergism regarding AKT activation based on pSer473‐AKT expression in cervical cancer cells. Hypoxia‐PI3K/AKT‐mediated transcriptional repression of the HPV early oncoproteins E6 and E7 was described to induce a state of dormancy of cervical cancer cells^40^ supporting oxygen‐dependent reversible growth arrest and evasion from prosenescent chemotherapy.^5^, ^10^ Thus, in addition to Th17‐mediated activation of AKT signaling in normoxic cervical cancer cells,^21^ our results showed that Th17 cells also support maintenance of this pro‐tumorigenic pathway in hypoxic cervical cancer cells. Furthermore, Th17‐hypoxia interactions enhanced the expression of the hypoxia‐induced proteins carbonic anhydrase IX (CAIX), hexokinase II and SLC2A1 in 2D cultures and 3D spheroids. These hypoxia‐related factors support cancer cells to adapt to changes in oxygen concentration and metabolism.^7^, ^30^ Accordingly, we found that Th17‐hypoxia interactions favor enhanced glucose uptake of cervical cancer cells, a process described to support cancer cell migration.^32^

Hypoxic regions are supposed as drivers for metastases^7^ and the presence of Th17 cells in cervical cancers was associated with poor prognosis.^17^, ^21^ Here we demonstrated that Th17 cells support an EMT phenotype of hypoxic cervical cancer cells by reduced e‐cadherin and increased vimentin expression. Functionally, Th17‐hypoxia interactions favored proliferation, migration, and invasiveness of cervical cancer cells. Previously, in experiments with 21% oxygen, we described that Th17 cells interfere with cervical cancer metabolism via miR‐142‐5p‐mediated suppression of the subunits C and D from the succinate‐dehydrogenase (SDH) complex, promoting cancer cell invasiveness and metastases.^26^ Thus, results from this study indicated that Th17 cells not only favor invasiveness of cervical cancer cells in more oxygenated regions of tumors, but they further support migration of hypoxic cervical cancer cells described to be able to avoid chemotherapeutic drug‐induced damaging.^5^

In search for a responsible mediator of Th17‐hypoxia‐mediated enhanced migration of cervical cancer cells, we identified the IGF2BP2 that belongs to the family of RNA‐binding proteins. While the expression of IGF2BP2 is low or absent in most human tissues,^41^ it is highly expressed in several tumor entities.^42^, ^43^, ^44^ IGF2BP2 is described as a regulator of glycolytic enzymes, like SLC2A1, and dedicated to affect glycolysis rate^33^ and regulator of EMT‐related factors^34^ driving metastases.^45^ In normoxic cervical cancer cells, IGF2BP2 expression is regulated by the HPV16/18 oncoproteins E6/E7 favoring aerobic glycolysis and cancer progression.^43^ However, in hypoxic cervical cancers HPV16/18 E6/E7 expression is reduced^5^ whereby the IGF2BP2 inducers are missing. Interestingly, in our study we found that Th17 cells enhance the IGF2BP2 expression in hypoxic 2D cultures and 3D spheroids of cervical cancer cells. Th17 cells increased IGF2BP2 expression in cervical cancer cells, on the one hand after direct cell–cell contact by co‐cultures, on the other hand stronger by soluble factors that were sufficient for IGF2BP2 induction enhancing migration and invasiveness of 2D cultures and 3D spheroids. Neutralization experiments identified Th17‐derived IL‐17 as the responsible inducer of IGF2BP2. This is in line with our previous findings identifying Th17‐produced IL‐17 as the responsible soluble factor that mediates resistance toward chemoradiotherapy^21^ or favored progression of cervical cancer cells.^25^ Our knock‐down of IGF2BP2 via siRNA reduced the Th17‐induced proliferation and migration of 2D‐cultured cervical cancer cells under hypoxic conditions. Additionally, we validated the role of IGF2BP2 for Th17‐induced invasiveness in 3D spheroids using recently described small molecule IGF2BP2 inhibitors from the benzamidobenzoic acid class.^27^ These compounds affected colorectal tumor growth in vivo in a zebrafish embryo xenograft model.^27^ Here, treatment of Th17‐instructed spheroids with these compounds significantly decreased the invasive behavior of 3D‐cultured cervical cancer cells underlining the role of Th17‐instructed IGF2BP2 expression for cervical cancer invasiveness. Thus, although hypoxia suppressed important regulators of IGF2BP2 in cervical cancers, our data showed that Th17 cells maintained the IGF2BP2 expression under hypoxic conditions favoring cervical cancer cell migration and invasive behavior.

Clinically most important, retrospective in situ analyses from this study further supported the in vivo relevance of our findings. The analyzed cervical cancer biopsies showed a heterogeneous expression of IGF2BP2 from negative to strong, and enhanced expression of IGF2BP2 correlated with lymph node metastases and cervical cancer recurrence. Interestingly, enhanced numbers of Th17 cells correlated with increased IGF2BP2 expression in situ, supporting our in vitro findings. Both parameters, IGF2BP2 expression at different intensities as well as Th17 numbers/mm^2^ > 10, were associated with reduced recurrence‐free survival. Notably, the combination of both factors, IRS of IGF2BP2 ≥ 3 and Th17 numbers/mm^2^ ≥ 10, led to the 3.5‐ and 7‐year recurrence‐free survival rate of 50% and 5.6%, respectively. In conclusion, numbers of Th17 cells^18^ as well as IGF2BP2 expression (this study) are increased in cervical cancers and are both associated with severity of disease.

Individual therapy responses driving cancer metastases and relapse are still a clinical problem, indicating the need for new (immune)therapeutic approaches. So far, two antibodies are approved as targeted immunotherapies: bevacizumab, an anti‐VEGF antibody, to inhibit angiogenesis in combination with a platinum‐based chemotherapy in patients with advanced, metastatic or recurrent cervical cancer^46^ as well as pembrolizumab, an anti‐PD‐1 antibody, for patients with high‐risk (FIGO 2014 stage IB2–IIB with node‐positive disease or stage III–IVA), locally advanced, histologically confirmed cervical cancer in combination with chemoradiotherapy.^47^ The results from this study highlight the relevance of Th17 cells as well as IGF2BP2 for cervical cancer progression and suggest this axis as a potential biomarker or target for therapy. First small inhibitors for IGF2BP2 were recently tested to treat colorectal cancer cells^27^ or T‐cell acute lymphoblastic leukemia.^48^ Based on our study, Th17 cell numbers in situ as initiators of the novel mechanism should be considered as a target for immunotherapy. Antibodies against IL‐17A or IL‐17 receptor are approved for the treatment of psoriasis^49^ and under consideration for the treatment of inflammatory diseases^50^ and cancers.^51^ In conclusion, our study identified a novel role of Th17 cells in cervical cancer progression. We could clarify that Th17 cells synergize with hypoxia favoring cervical cancer migration and invasiveness in an IGF2BP2‐dependent manner. In accordance with our previous results,^26^ data from this study indicate that Th17 cells favor migration and invasion of cervical cancer cells in the presence of higher and lower oxygen concentration. This may explain the association of Th17 cells in cervical cancers with poor prognosis supporting the idea of Th17‐based immunotherapeutic approaches in cancer therapy.

## AUTHOR CONTRIBUTIONS

**Selina Gies:** Conceptualization; methodology; investigation; validation; formal analysis; supervision; visualization; writing – original draft; writing – review and editing; data curation. **Maike Pohlers:** Methodology; investigation; writing – review and editing. **Tanja Tänzer:** Methodology; investigation. **Emmanuel Ampofo:** Resources. **Matthias W. Laschke:** Writing – review and editing; resources. **Moritz Schäfer:** Methodology; investigation; writing – review and editing. **Yoo‐Jin Kim:** Resources; writing – review and editing; formal analysis. **Rainer Maria Bohle:** Resources; writing – review and editing. **Erich‐Franz Solomayer:** Resources; writing – review and editing. **Konrad Wagner:** Methodology; resources. **Martin Empting:** Resources; writing – review and editing. **Alexandra K. Kiemer:** Resources; writing – review and editing. **Barbara Walch‐Rückheim:** Conceptualization; methodology; data curation; investigation; validation; formal analysis; supervision; funding acquisition; visualization; project administration; resources; writing – original draft; writing – review and editing.

## FUNDING INFORMATION

This work was supported by a grant from the ‘Dr. Rolf Schwiete Stiftung’ (2023‐012) and a grant of the ‘Saarland University’ to B. Walch‐Rückheim.

## CONFLICT OF INTEREST STATEMENT

The authors declare no potential conflicts of interest.

## ETHICS STATEMENT

This study has been conducted according to Declaration of Helsinki principles. The protocols for IHC stainings of anonymized tissue samples were approved by the Ethics Committees of the Saarland Ärztekammer (Saarbrücken, Germany; 98/17). Written informed consent was provided by all study participants.

## Supporting information


**Data S1.** Supporting Information.
