# Supplementary material for: Th17 cells favor migration and invasiveness of cervical cancer cells under hypoxia in an IGF2BP2‐dependent manner
Source: Int J Cancer. 2026 Jan 24;158(11):3021–37. doi: 10.1002/ijc.70340 (PMC13047252; doi:10.1002/ijc.70340)
Supplement: Supplementary file 1 — Data S1. Supporting Information. [file IJC-158-3021-s001.pdf]

Th17 cells favor migration and invasiveness of cervical cancer cells under hypoxia in an IGF2BP2-dependent manner

Selina Gies, Maike Pohlers, Tanja Tänzer, Emmanuel Ampofo Matthias W. Laschke, Moritz Schäfer, Yoo-Jin Kim, Rainer Maria Bohle, Erich-Franz Solomayer, Konrad Wagner, Martin Empting, Alexandra K. Kiemer, Barbara Walch-Rückheim

Table of contents:

Supplementary Materials and Methods

Supplementary Figure S1: Th17-induced expression of pAKTSer on protein level under hypoxic conditions.

Supplementary Figure S2: HIF-1α expression after incubation of the cells by 1% oxygen.

Supplementary Figure S3: Th17-regulated vimentin and e-cadherin expression on mRNA and protein level under hypoxic conditions.

Supplementary Figure S4: Cobalt chloride induced HIF-1α expression.

Supplementary Figure S5: Th17 cells induced the expression of IGF2BP2 in cervical cancer cells on mRNA and protein level.

Supplementary Figure S6: ROC analysis of IGF2BP2 expression and Th17 numbers.

Supplementary Table S1: Immunoreactive Score (IRS) according to Remmele & Stegner.

Supplementary Materials and Methods

Cell culture

Cells were cultured in DMEM (Sigma Aldrich, Taufkirchen, Germany) supplemented with 10% heat-inactivated endotoxin tested fetal calf serum (FCS, Gibco), 1 mM sodium pyruvate, and 2 mM L-alanyl-L-glutamine. Mycoplasma testing was performed monthly using Mycotrace PCR detection kit (PAA, Heidelberg, Germany) or Venor®GeM Classic (Minerva Biolabs; Berlin, Germany).

Quantitative real-time PCR

The 94-bp fragment of RPL13A was detected with primers 5'-AGCGGATGAACACCAACC-3' and 5`-TTTGTGGGGCAGCATACTC-3' and probe no. 28; the 105-bp fragment of CAIX was detected with primers 5'-CCTTTGCCAGAGTTGACGAG-3' and 5'-GCAACTGCTCATAGGCACTGT-3' and probe no. 25; the 66-bp fragment of Hexokinase II was detected with primers 5'-TCCCCTGCCACCAGACTA-3' and 5'-TGGACTTGAATCCCTTGGTC-3' and probe no. 54; the 113-bp fragment of SLC2A1 was detected with primers 5'-GCCCATGTATGTGGGTGAA-3' and 5'-AGTCCAGGCCGAACACCT-3' and probe no. 81; the 80-bp fragment of IGF2BP2 was detected with primers 5'-TTCTGGTCGGGGTAGTCCAC-3' and 5'-TTTGGGGACAGGAAGCTGC-3' and probe no. 18; the 113-bp fragment of Vimentin was detected with primers 5'-GAGGGAGTGAATCCAGATTAGTTT-3' and 5'-TGAGATTGCCACCTACAGGAA-3' and probe no. 11 and the 95-bp fragment of E-Cadherin was detected with primers 5'-GATGGCGGCATTGTAGGT-3' and 5'-GGTCTGTCATGGAAGGTGCT-3' and probe no. 5.

siRNA transfections

2x10^5 (SiHa) and 2.5x10^5 (SW756) cells/12-well were transfected with Lipofectamine RNAi-Max (Lipofect RNAi Max) and 30 pmol of IGF2BP2 specific siRNA (human siRNA IGF2BP2 #1: GCUAAGCGGGCAUCAGUUU, human siRNA IGF2BP2 #2: GAGCAUAUACAACCCGGAA; Dharmacon, Horizon, Cambridge, UK) or 30 pmol of siControl (NON-Target #2 (D-001810-02-20); Horizon). SiRNA-mediated knock down was confirmed by qPCR and western blot analysis.

Immunofluorescence of 3D spheroids

Five-µm-thick sections of paraffin-embedded spheroids on silane-coated slides (Engelbrecht, Edermünde, Germany) were used. For IF, antigen retrieval was performed by heating the sections in TE buffer (10 mM Tris, 1 mm EDTA pH 9.0) at 95 °C for 15 min using microwave and immersed in 3% H_2O_2 in TBS (50 mM Tris-HCl, 150 mM NaCl, pH 7.6) to block endogenous peroxidase activity. To reduce non-specific staining, each section was treated with 2.5% normal horse serum for 30 min. Antibodies against CAIX (rabbit, 1:10.000, Abcam), SLC2A1 (rabbit, 1:5.000, CellSignaling), Hexokinase II (rabbit, 1:1000, CellSignaling) and IGF2BP2 (rabbit, 1:200, Sigma-Aldrich) were used and TSA™ Kit #13, with HRP-Goat Anti-Rabbit IgG and Alexa Fluor® 546 Tyramide (Life Technology, Darmstadt, Germany) according to the manufacturer's instructions. For nuclear staining, 1 µg/ml of DAPI (4′,6′-diamidino-2-phenylindole) (Sigma Aldrich) was used.

Tissue specimens, IHC, and IF analysis

Two-micrometer thick formalin-fixed paraffin-embedded sections fixed on microslides were deparaffinized with xylene and hydrated using a diluted alcohol series. For immunohistochemistry or IF, sections were incubated with TE buffer (10 mM Tris, 1 mM EDTA, pH 9.0) and cooked for 20 min using microwave, respectively, for antigen retrieval and immersed in 3% H_2O_2 in TBS (50 mM Tris/HCl, 150 mM NaCl, pH 7.6) to block endogenous

peroxidase activity. To reduce nonspecific staining, each section was treated with 2.5% normal horse serum for 30 min. Rabbit anti-IGF2BP2 antibody (1 : 100; AB_2863480; Sigma-Aldrich), rabbit anti-CAIX (1:1000, AB_10861549, Abcam), mouse anti-CD4 monoclonal antibody 4B12 (1 : 1000; Leica Biosystems; AB_563560), and rabbit anti-IL-17 polyclonal antibody (1 : 500; Abcam; AB_1603584) were used. For immunohistochemistry, ImmPRESS Detection Kit (Vector Laboratories, Burlingame, CA, USA) was used. For IF TSA™ Kit #2, with HRP-Goat Anti-Mouse IgG and Alexa Fluor™ 488 Tyramide and TSA™ Kit #13, with HRP-Goat Anti-Rabbit IgG and Alexa Fluor® 546 Tyramide (Life Technology) were used according to the manufacturer‘s instructions.

Table 1: List of used materials.

**ANTIBODIES**			
**Antigen/Protein**	**Dilution/ concentration**	**Vendor**	**Identifier**
rabbit anti CAIX	1:200 for IF; 1:1000 for IHC; Fig. 2A, D, Fig. 6E	Abcam	#ab108351 RRID:AB_10861549
rabbit anti SLC2A1	1:200 for IF; Fig. 2B, E	Cell Signaling	#73015 RRID:AB_3064908
Goat anti-rabbit IgG (H+L) Highly Cross-Adsorbed Secondary Antibody, Alexa Fluor™ 546	1:200 for IF; Fig. 2A, 2B, S2, S4	Invitrogen	#A-11035 RRID: AB_2534093
rabbit anti hexokinase	1:1000 for WB; Fig. 2C, 1:1000 for IF; Fig. 2F	Cell Signaling	#2867 RRID:AB_2232946
mouse anti β-actin, clone AC-15	1:5000 for WB; Fig. 2C, 4B, 5A, S1, S3B	Sigma-Aldrich,	#A5441 RRID:AB_476744
rabbit anti IGF2BP2	1:1000 for WB; Fig. 4B, 1:200 for IF; Fig. 4C, 1:100 for IHC; Fig. 6A	Sigma-Aldrich	#SAB5701598 RRID:AB_2863480
rabbit anti IL-17 polyclonal antibody	1:1000 for IF; Fig. 6	Abcam	#ab79056 RRID:AB_1603584
mouse anti CD4 monoclonal antibody, clone 4B12,	1:500 for IF; Fig. 6	Leica Biosystems,	#CD4-368-L-CE-H RRID:AB_563560
rabbit anti pAKTSer	1:2000 for WB; Fig. S1	Cell Signaling	#4060 RRID:AB_2315049
rabbit anti HIF-1α	1:200 for IF; Fig. S2A, S4A	Invitrogen	#PA1-16601 RRID:AB_2117128
mouse anti e-cadherin	1:1000 for WB; Fig. S4B	Cell Signaling	#14472 RRID: AB_2728770
rabbit anti vimentin	1:1000 for WB; Fig. S4B	Cell Signaling	#5741 RRID: AB_10695459
anti mouse IgG-POX	1:5000 for WB	Sigma Aldrich	#A9044 RRID:AB_258431
anti rabbit IgG-POX	1:5000 for WB	Sigma Aldrich	#A0545 RRID:AB_257896

CHEMICALS, PEPTIDES, AND RECOMBINANT PROTEINS		
Human IL-17, research grade	Miltenyi Biotec	#130-094-625
Human IL-6	Miltenyi Biotec	#130-095-365
Human TGF-β1, premium grade	Miltenyi Biotec	#130-095-067
Human IL-1β	Miltenyi Biotec	130-093-895
Human IL-23, research grade	Miltenyi Biotec	#130-095-757
RPMI-1640 medium	Sigma-Aldrich	#R8758
Dulbecco's Modified Eagle's Medium - high glucose	Sigma-Aldrich	#D5796
Dulbecco's Phosphate Buffered Saline	Sigma-Aldrich	#D8537
Fetal Bovine Serum	Sigma-Aldrich	#F7524
Fetal Bovine Serum, qualified (-LPS)	Gibco	#10270-106
Trypsin-EDTA	Sigma-Aldrich	#T3924
Sodium pyruvate solution	Sigma-Aldrich	#S8636
Trypan-Blau	Sigma-Aldrich	#T6146
UltraPure EDTA	GIbco	#15575
Amersham™ Protran® Western blotting membranes, nitrocellulose	GE Healthcare Life Science	#10600002
Amersham™ Hybond® SEQ Western blotting membranes, PVDF	GE Healthcare Life Science	#10600023
Blotting Paper Sheets	neoLab	#2-4322
Methanol	VWR	#20847320
Pierce Prestained Protein Molecular Weight Marker	ThermoFisher Scientific	#26612
Ponceau	Sigma-Aldrich	#P-3504
Acetic acid	Merck	#64-19-7
TEMED	AppliChem	#A1148,0100
Rotiphorese	Roth	#3029.1
APS	AppliChem	#A2941,0100
Mercaptopropandiol	Sigma-Aldrich	#8417050010
SDS	AppliChem	#1423631211
Glycerin	Merck	#1.04093.1000
QIAzol Lysis Reagent	QIAGEN	#79306
Chloroform	Sigma-Aldrich	#24216
Ethanol	Th.Geyer	#2273100
Isopropanol	VWR	#20842330
DEPC water	Invitrogen	#10977-035
peqGold dNTP-Set	peqLab	#20-2011
Random Hexamer Primer	Merck	#ST05357629-010
RNAse Inhibitor (RiboLock)	Thermo scientific	#EO0381
BSA	AppliChem	#A1391.0100
Powdered milk	Serva	#42590.03
Tween	Chemsolute	#80220500
DMSO	AppliChem	#A3672,0250
BSA	ThermoSctientific	#B14
A.dest	Braun	#6724092.00.00
Salmon Sperm DNA	Invitrogen	#15632-011
TE-EDTA	Sigma-Aldrich	#93283
Opti-MEM™, GlutaMAX™ I	Thermo Fisher	#51985034
Lipofectamine™ RNAiMAX	Thermo Fisher	#13778150
5x siRNA buffer	Dharmacon	#B-002000-UB-100
Pancoll human	PAN biotech	#P04-601000
Matrigel® Growth Factor Reduced (GFR) Basement Membrane Matrix	Corning Costar Corp	#354230
Agarose	Sigma-Aldrich, Low EEO	#A6877, #A2114,0500

BSA 7,5%	Sigma-Aldrich	#A8412
EGF	Peprotech	#AF-100-15
FGFbasic	Peprotech	#AF-100-18B
Insulin	Fisher	#11508856
B27	Fisher	#17504044
Dulbecco's Modified Eagle's Medium F12 - high glucose	Gibco	# 31330-038
Paraformaldehyde	Sigma-Aldrich	#P6148
TritonX100	AppliChem	#A4975,0500
BSA in blocking solution	Life technologiesTM	#T20923
DAPI	Sigma-Aldrich	#D9542
Tyramid working solution AlexaFluor546	Invitrogen	#B40954
Amplification buffer	Life technologiesTM	#T20923
Eosin	Merck	#1041340025
glacial acetic acid	Merck	#PHR1748
Hematoxylin	Sigma-Aldrich	#MHS16
Xylol	Roth	#2662.1
EDTA	Merck	#108418
Hydrogen peroxidase	Chemsolute	#4761011
Permanent Mounting Medium (VectaMount)	Vector Laboratories	#H-5000
Antifade Mounting Medium (Vecta Shield)	Vector Laboratories	#H-1000
Cobaldchloride	Sigma-Aldrich	#15862
NBDG	Thermo scientific, Invitrogen	#N13195
silane-coated slides	Engelbrecht	#111252A
IGF2BP2 inhibitors	Dahlem et al., 2022; DOI: 10.1021/acschembio.1c00833	
**COMMERCIAL ASSAYS**		
Maxima Reverse Transcriptase	ThermoFisher Scientific	#EP0742
Fast Start Taq DNA Polymerase dNTPack	Roche	#4738357001
ImmPRESS® HRP Horse Anti-Rabbit IgG Polymer Detection Kit, Peroxidase	Vector Laboratories	#MP-7401
Invitrogen™ Molecular Probes™ TSA™ Kit 13, with HRP-Goat Anti-Rabbit IgG and Alexa Fluor™ 546 Tyramide	Life Technology	#T20923
Impact DAB Diluent + DAB Chromogen	Vector Laboratories	#SK-4105
Naive $CD4^+$ T Cell Isolation Kit II, human	Miltenyi Biotec	#130-094-131
IL-17 Secretion Assay – Cell Enrichment and Detection Kit (PE), human	Miltenyi Biotec	#130-094-542
T Cell Activation/Expansion Kit (MACSi Beads)	Miltenyi Biotec	#130-091-441
SuperSignal West Dura Substrate	Thermo Fisher	#34076
Human IL-17 DuoSet ELISA	R&D Systems	#DY317
DuoSet ELISA Ancillary Reagent Kit 2	R&D Systems	#DY008
Venor®GeM Classic	Minerva Biolabs	#11-1025
**EXPERIMENTAL MODELS: CELL LINES**		
Human HPV-16 positive cervical cancer cell line SiHa	ATCC	CVCL_0032
Human HPV-18 positive cervical cancer cell line HeLa	ATCC	CVCL_0030
Human HPV-18 positive cervical cancer cell line SW756	ATCC	CVCL_1727
**OLIGONUCLEOTIDES**		
ON-TARGETplus Non-targeting siRNA #2; targeting sequence: UGGUUUACAUGUUGUGUGA	Horizon Discovery	D-001810-02
ON-TARGETplus siRNA IGF2BP2 #07, targeting sequence: GCUAAGCGGGCAUCAGUUU	Horizon Discovery	SO-3155581G
ON-TARGETplus siRNA IGF2BP2 #08, targeting sequence: GAGCAUAUACAACCCGGAA	Horizon Discovery	SO-3155581G

qRTPCR primer for human specific CAIX<br>Forward 5`-3`: CCTTTGCCAGAGTTGACGAG<br>Reverse 5`-3`: GCAACTGCTCATAGGCACTGT	This study	Sigma Aldrich
qRTPCR primer for human specific Hexokinase<br>Forward 5`-3`: TCCCCTGCCACCAGACTA<br>Reverse 5`-3`: TGGACTTGAATCCCTTGGTC	This study	Sigma Aldrich
qRTPCR primer for human specific SLC2A1<br>Forward 5`-3`: GCCCATGTATGTGGGTGAA<br>Reverse 5`-3`: AGTCCAGGCCGAACACCT	This study	Sigma Aldrich
qRTPCR primer for human specific IGF2BP2<br>Forward 5`-3`: TTTGGGGACAGGAAGCTGC<br>Reverse 5`-3`: TTCTGGTCGGGGTAGTCCAC	This study	Sigma Aldrich
qRTPCR primer for human specific CDH1<br>Forward 5`-3`: GATGGCGGCATTGTAGGT<br>Reverse 5`-3`: GCTCTGTCATGGAAGGTGCT	This study	Sigma Aldrich
qRTPCR primer for human specific VIM<br>Forward 5`-3`: TGAGATTGCCACCTACAGGAA<br>Reverse 5`-3`: GAGGGAGTGAATCCAGATTAGTTT	This study	Sigma Aldrich
qRTPCR primer for RPL13A<br>Forward 5`-3`: AGCGGATGAACACCAACC<br>Reverse 5`-3`: TTTGTGGGGCAGCATACTC	This study	Sigma Aldrich
**SOFTWARE**		
Image Lab	Bio Rad	https://www.bio-rad.com/de-de/product/image-lab-software
Image Composite Editor	Microsoft	https://www.microsoft.com/en-us/research/project/image-composite-editor/
VIS (Visiopharm Integrator sytem)	Visiopharm	https://visiopharm.com/
cellSens Dimension	Olympus	https://www.olympus-lifescience.com/de/software/cellsens/
GraphPad Prism8	GraphPad	https://www.graphpad.com/scientific-software/prism/
ZEN3.1	Zeiss	https://www.mikroskop-center.de/zen-3-1-pro

Supplementary Table S1: Immunoreactive Score (IRS) according to Remmele & Stegner.

A (% of positive cells)	B (Staining intensity)	A x B= IRS
0 = no positive cells	0 = no positive cells	0-2 = negative
1 = <10% of positive cells	1 = weak color reaction	3-4 = weak
2 = 10-50% of positive cells	2 = moderate color reaction	6-8 = moderate
3 = 51-80% of positive cells	3 = strong color reaction	9-12 = strong
4 = >80% positive cells		

Figure S1

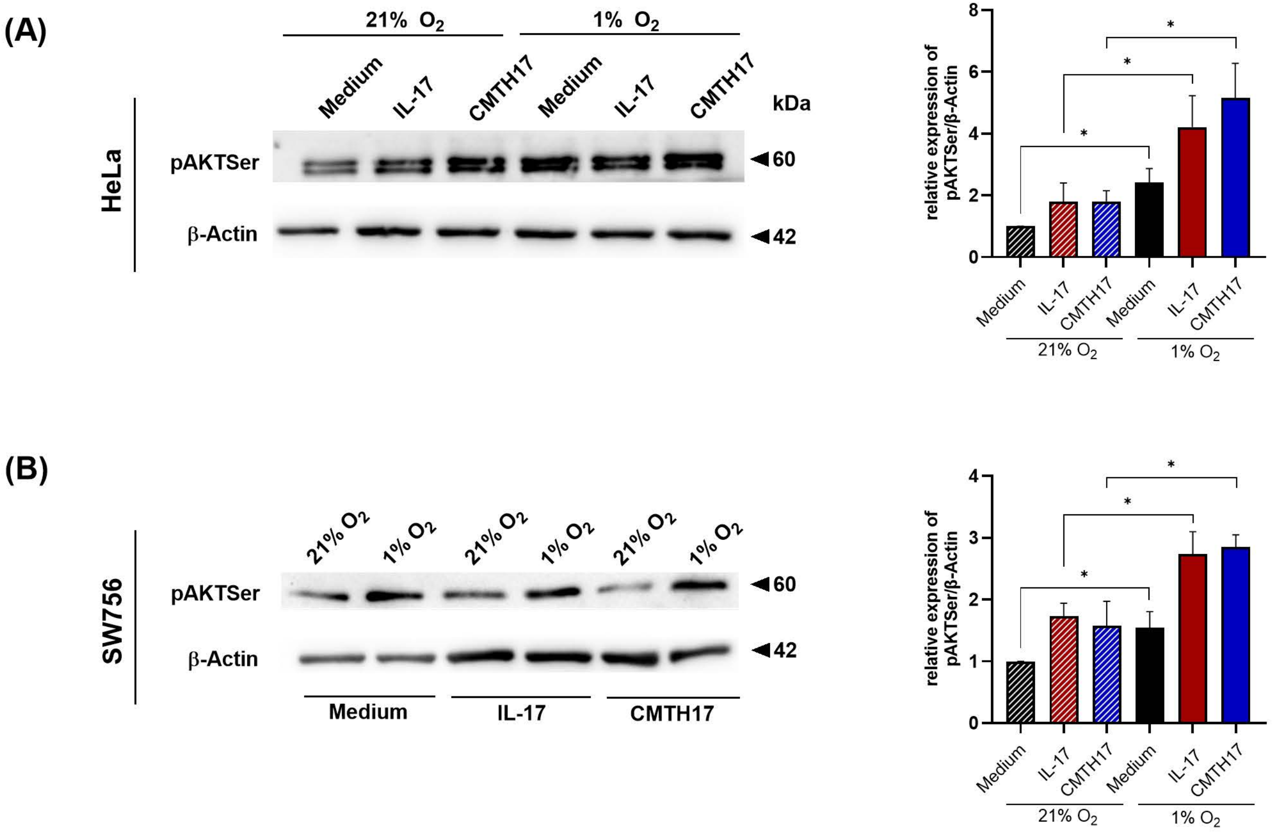

Figure S1: Th17-induced expression of pAKTSer on protein level under hypoxic conditions. HeLa (A) or SW756 cells (B) were stimulated with 100 ng/ml rhIL-17 or 20% CMTH17 for 24 h by normoxic (21% oxygen) or hypoxic conditions (1% oxygen). Whole cell extracts were analyzed for pAKTSer expression by western blot analysis. β-Actin was used as a loading control. The relative pAKTSer expression (pAKTSer/ β-Actin) in cells incubated by 21% oxygen was set at 1. Depicted is one out of four independent western blots. Bars represent quantification of n=4 experiments. P-value according to the nonparametric Mann-Whitney U-test. Asterisks (*p<0.05) represent statistical significances.

Figure S2

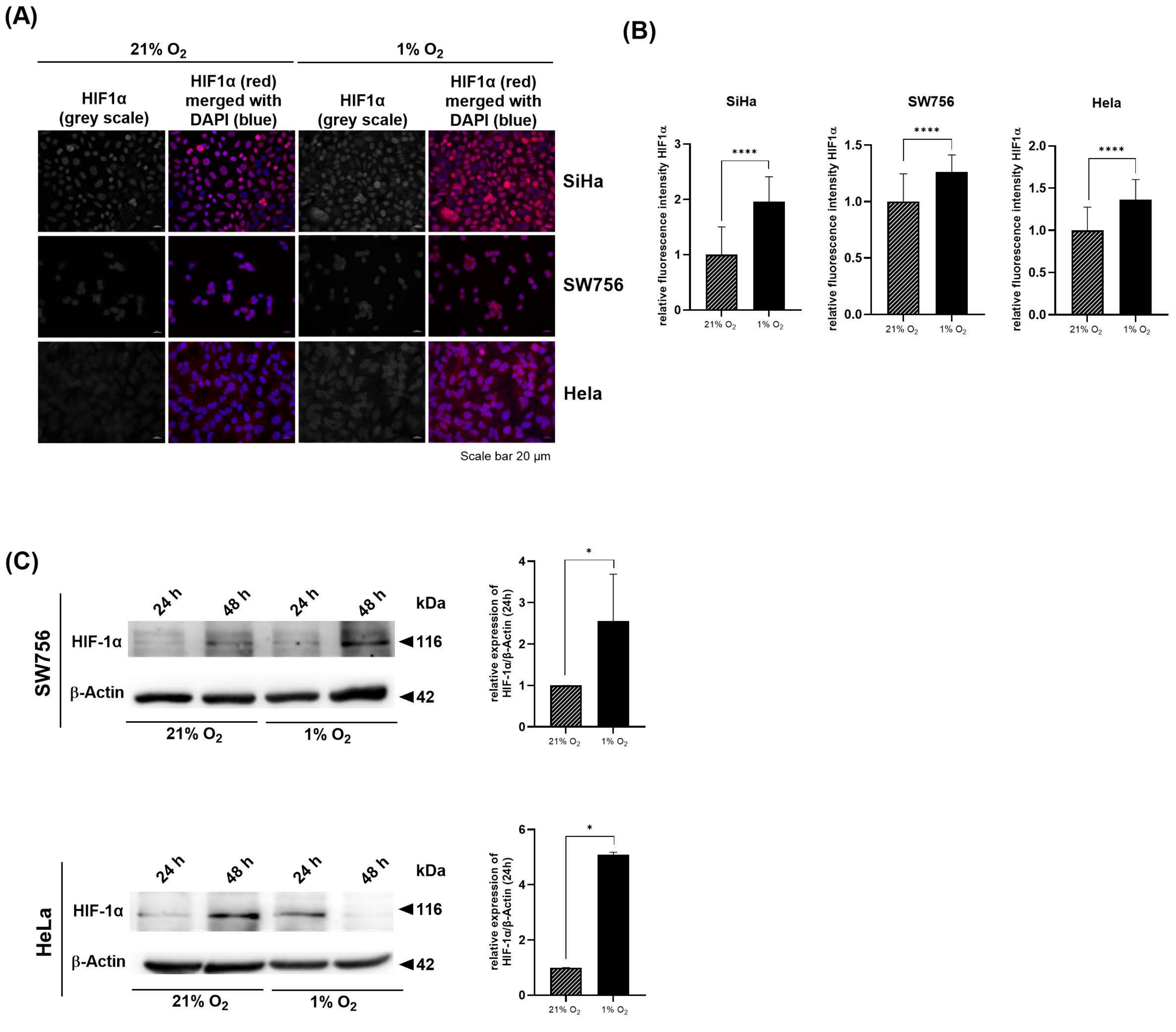

Figure S2: HIF-1α expression after incubation of the cells by 1% oxygen. (A) 2D monolayers of SiHa, SW756 and HeLa were incubated by hypoxic conditions (1% O_2) or normoxic (21% O_2) conditions. 24 hours later, HIF-1α expression was investigated by IF. (B) Bars represent quantification of relative fluorescence/10 cells of 20 independent pictures (mean + SD, 10 cells/ picture were measured, magnification 400x, Scale bar: 20 µm) from n=1 independent experiments performed in doubles. The values of medium stimulated cells (black stripped bars, 21% oxygen) were set at 1. (C) SW756 or HeLa cells were incubated hypoxic (1% oxygen) or normoxic (21% oxygen). After 24h or 48h whole cell extracts were analyzed for HIF-1α expression by western blot analysis. β-Actin was used as a loading control. The relative HIF-1α expression (HIF-1α/ β-Actin) in cells incubated by 21% oxygen after 24h was set at 1. Depicted is one out of four independent western blots. Bars represent quantification of n=4 experiments. P-value according to the nonparametric Mann-Whitney U-test. Asterisks (*$p<0.05$; ****$p<0.0001$) represent statistical significances.

Figure S3

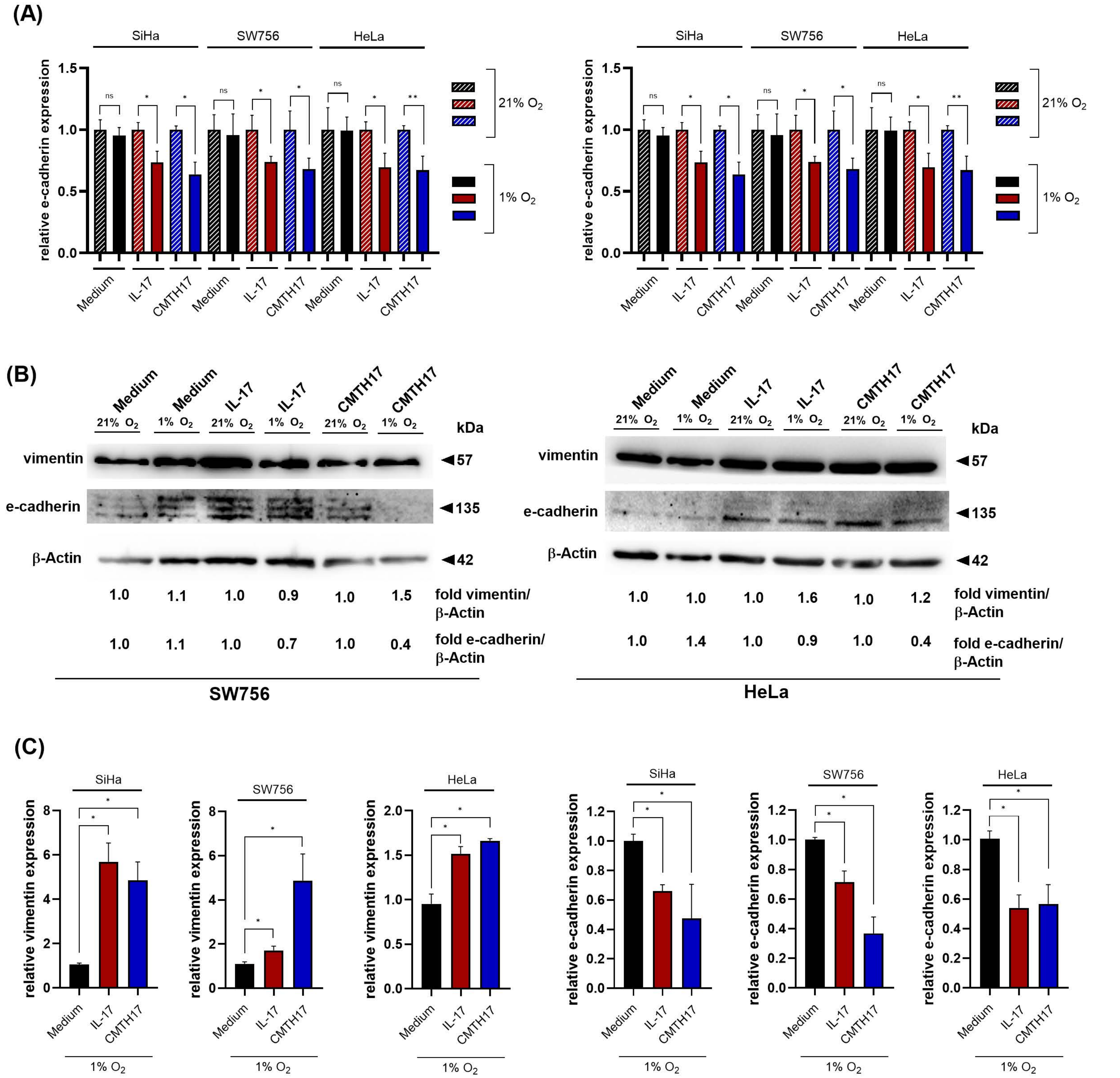

Figure S3: Th17-regulated vimentin and e-cadherin expression on mRNA and protein level under hypoxic conditions. (A) SiHa, SW756 and HeLa cells were stimulated with medium (black bars), IL-17 (100 ng/ml, red bars) or conditioned media of *in vitro* generated Th17 cells (CMTH17, 20%, blue bars) and incubated by normoxic (21% O_2, striped bars) or hypoxic oxygen conditions (1% O_2). After 24 h, cells were analyzed for vimentin and e-cadherin expression by qRT-PCR analysis. The quotient of the gen of interest/RPL13A of medium, IL-17 and CMTH17 stimulated cells which were incubated by 21% oxygen was set at 1. Shown are the results (mean ±SD) from three independent experiments performed in doubles. (B) SW756 and HeLa cells were stimulated with 100 ng/ml rhIL-17 or 20% CMTH17 for 24 h by normoxic or hypoxic conditions. Whole cell extracts were analyzed for vimentin and e-cadherin expression by western blot analysis. The relative vimentin and e-cadherin expression (vim/ β-Actin; e-cad/ β-Actin) in cells incubated by 21% oxygen was set at 1. β-Actin was used as a loading control. (C) SiHa, SW756 and HeLa cells were stimulated with medium (black bars), IL-17 (100 ng/ml, red bars) or conditioned media of *in vitro* generated Th17 cells (CMTH17, 20%, blue bars) and incubated by hypoxic oxygen conditions (1% O_2). After 72 h, cells were analyzed for vimentin and e-cadherin expression by qRT-PCR analysis. The quotient of the gen of interest/RPL13A of medium, IL-17 and CMTH17 stimulated cells which were incubated by 1% oxygen was set at 1. Shown are the results (mean ±SD) from four independent stimulations. Asterisks (*p<0.05; **p<0.01) represent statistical significances.

Figure S4

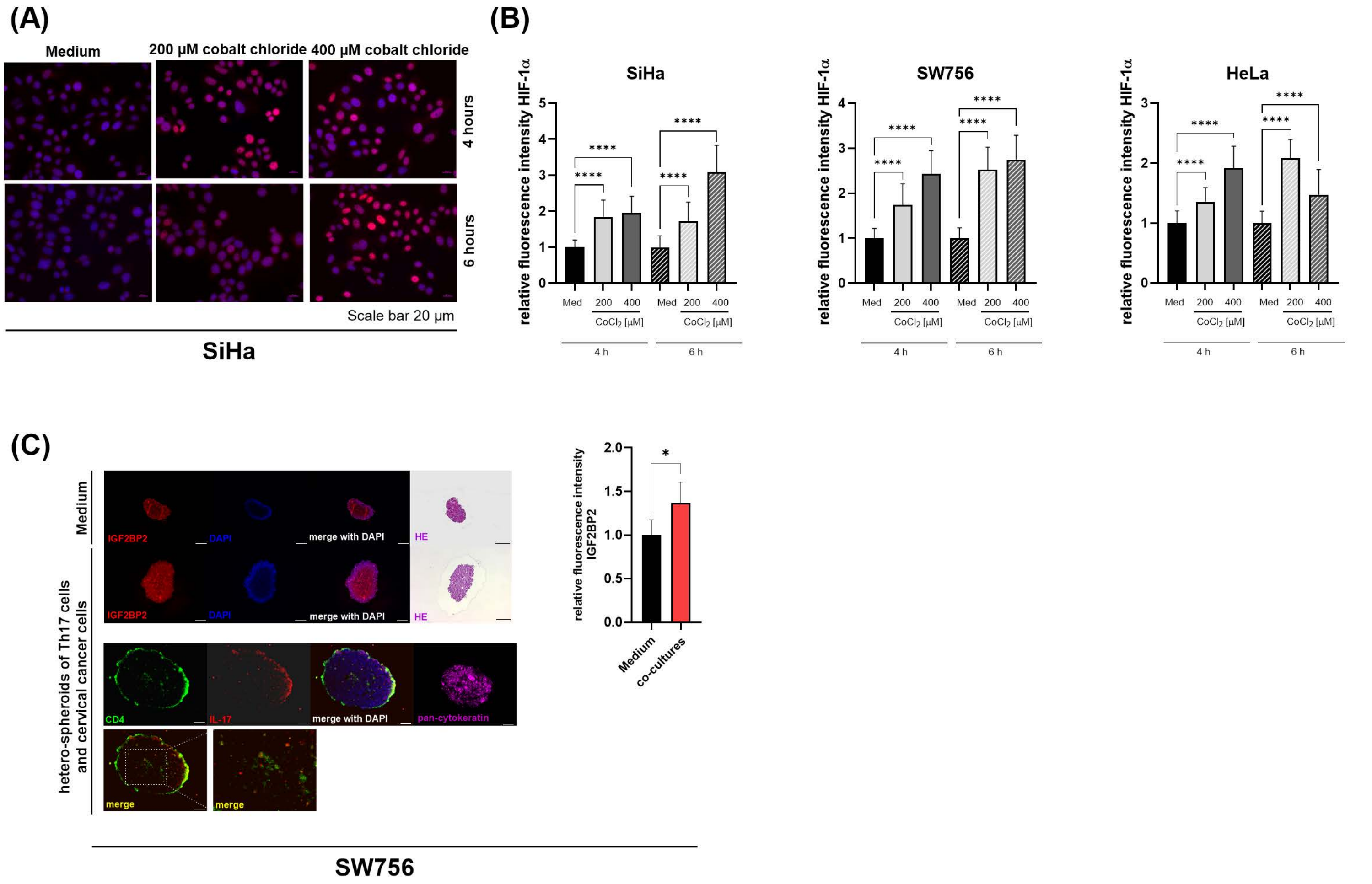

Figure S4: Cobalt chloride induced HIF-1α expression. (A, B) 2D monolayers of SiHa, SW756 and HeLa were stimulated with 200 or 400 µM cobalt chloride, respectively. 4 or 6 hours (striped bars) later, HIF-1α expression was investigated by IF (A). (B) Bars represent quantification of relative fluorescence/10 cells of 20 independent pictures (mean + SD, 10 cells/ picture were measured, magnification 400x, Scale bar: 20 µM) from n=1 experiment performed in doubles. The values of medium stimulated cells (B: black bars, non-stripped (4 h), stripped (6 h)) was set at 1. P-value according to the nonparametric Mann-Whitney U-test. Asterisks (****p<0.0001) represent statistical significances. (C) **Increased IGF2BP2 expression in hetero-spheroids of Th17 and cervical cancer cells.** 3D spheroids of SW756 cells and TH17 cells were generated over 10 days. 5 µm sections of fixed paraffin-emmbedded spheroids were validated by HE stainings and analyzed for IGF2BP2 expression (upper panel), CD4 or IL-17 expression (lower panel) by IF. Bars represent quantification of relative fluorescence/spheroid of IGF2BP2 expression of n=6 independent spheroids, respectively. P-value according to the nonparametric Mann-Whitney U-test. Asterisks represent statistical significances: *p<0,05.

Figure S5

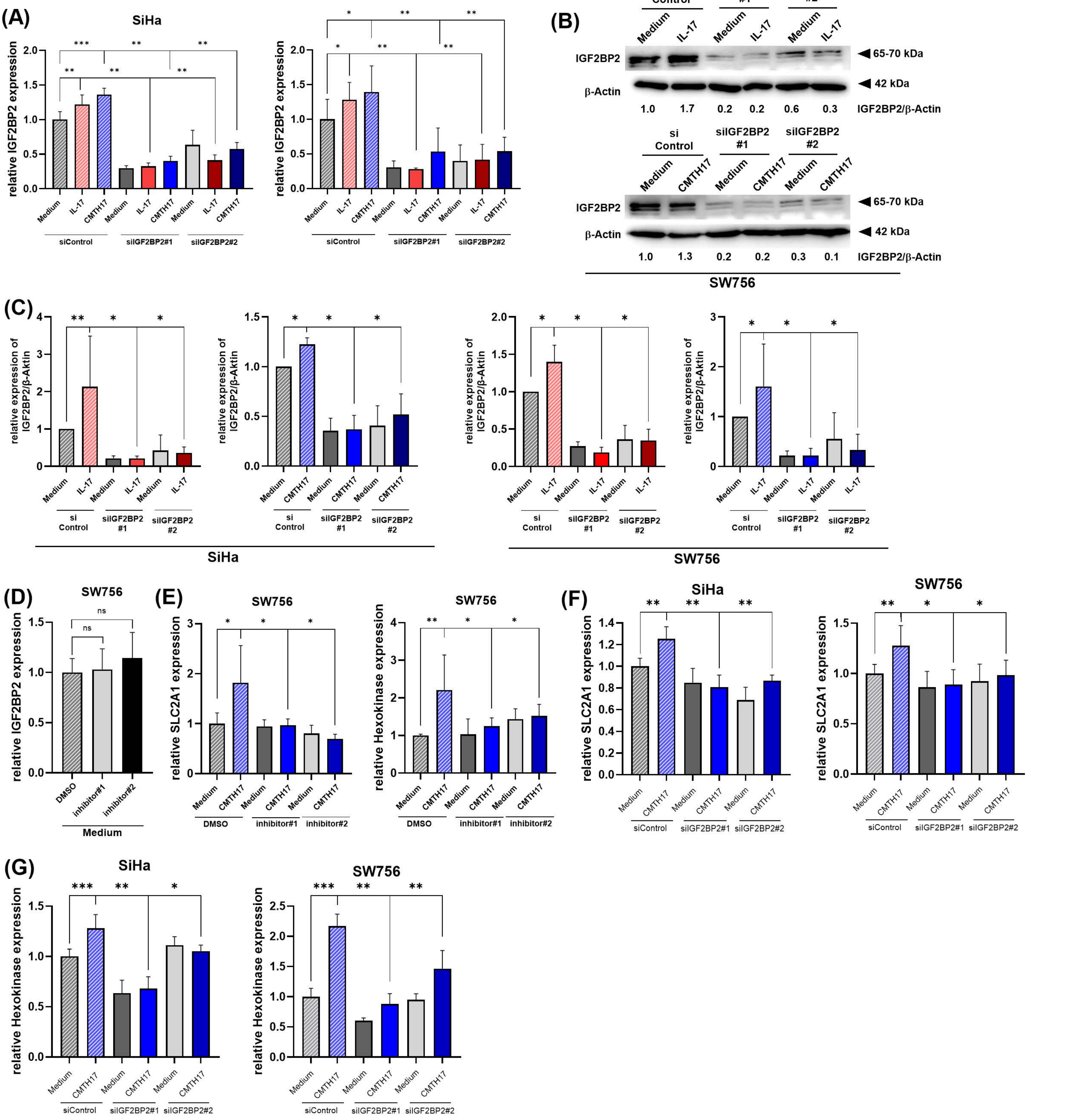

Figure S5: Th17 cells induced the expression of IGF2BP2 in cervical cancer cells on mRNA and protein level. (A) SiHa (left) and SW756 (right) were transfected with siRNAs (siControl, siIGF2BP2 #1, #2) and stimulated with 100 ng/ml rhIL-17 (red bars) or CMTH17 (blue bars) for 24 h under hypoxic conditions. After 24 h, cells were analyzed for IGF2BP2 expression by qRT-PCR analysis. The quotient of the gen of interest/RPL13A of medium stimulated cells which were transfected with non-target siRNA was set at 1. Shown are the results mean + SD from six independent stimulations. (B, C) (B) SW756 and (C) SW756 and SiHa cells were transfected with siRNAs (non-target, siIGF2BP2 #1, #2) and stimulated with 100 ng/ml rhIL-17 (red bars) or CMTH17 (blue bars) for 24 h under hypoxic conditions. Whole cell extracts were analyzed for IGF2BP2 expression by western blot analysis. β-Aktin was used as a loading control. (B) Depicted is one representative western blot (SW756). The relative IGF2BP2 expression (IGF2BP2/ β-Actin) of medium stimulated and non-target transfected cells was set at 1. Bars represent the results mean + SD from four independent stimulations. (D, E) SW756 were threatened with IGF2BP2 specific inhibitors (IGF2BP2 #1, #2) or DMSO as a control and stimulated with 300 µM cobalt chloride. After 24 h, cells were analyzed for (D) IGF2BP2 and (E) SLC2A1 expression by qRT-PCR analysis. The quotient of the gen of interest/RPL13A of medium stimulated cells which were threatened with DMSO was set at 1. Shown are the results mean + SD from two independent experiments performed in doubles. (F, G) SiHa (left) and SW756 (right) were transfected with siRNAs (non-target, siIGF2BP2 #1, #2) and stimulated with CMTH17 (blue bars) for 24 h under hypoxic conditions. After 24 h, cells were analyzed for (F) SLC2A1and (G) hexokinase II expression by qRT-PCR analysis. The quotient of the gen of interest/RPL13A of medium stimulated cells which were transfected with siControl was set at 1. Shown are the results mean + SD from six independent stimulations. P-value according to the nonparametric Mann-Whitney U-test. Asterisks represent statistical significances (*p<0,05, **p<0,01, ***p<0,001, ****p<0,0001, n.s.=not significant).

Figure S6

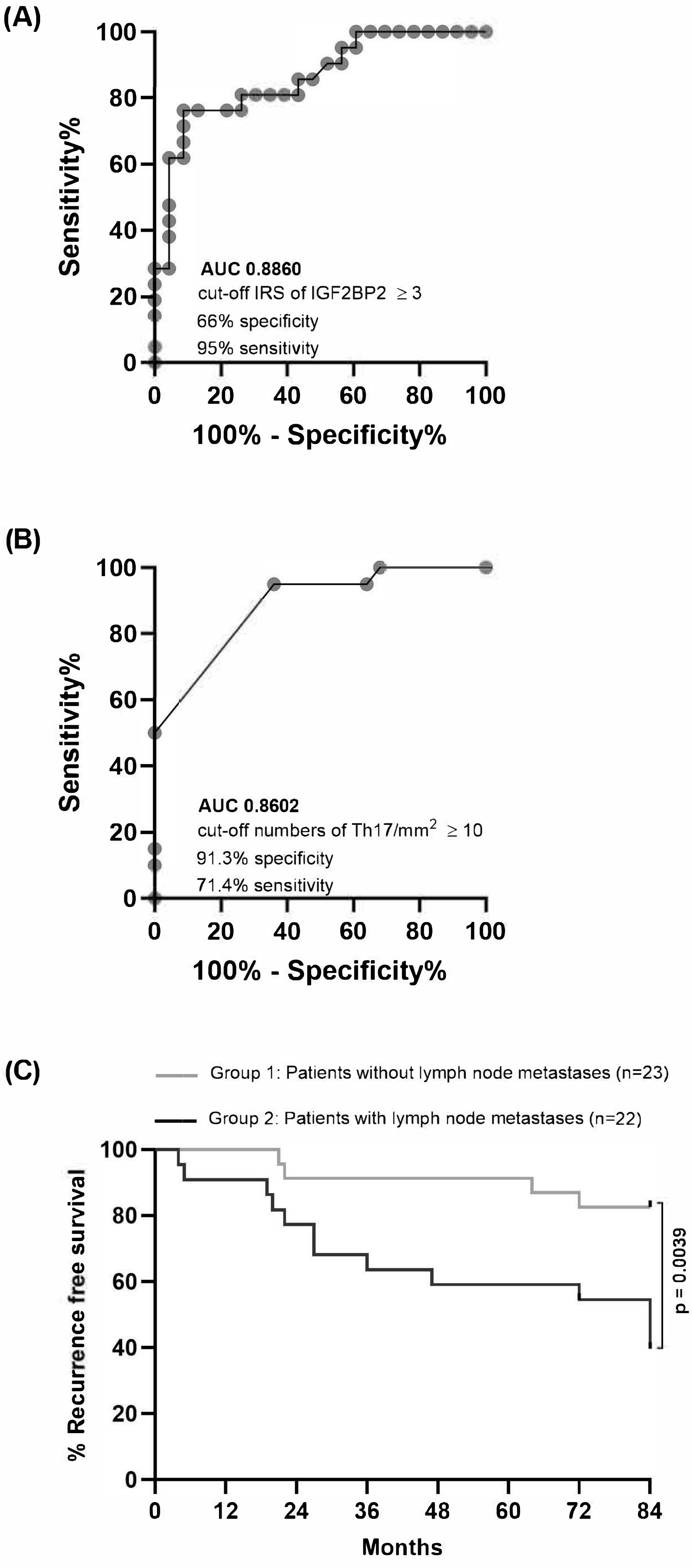

Figure S6: ROC analysis of IGF2BP2 expression and Th17 numbers. ROC analysis of (A) IRS of IGF2BP2 and (B) Th17 numbers/mm^2. (C) Recurrence-free survival of 45 patients was determined for a cohort with lymphnode metastases and without lymph node metastases. Median recurrence-free survival was 84 months for the cohort with lymph node metastases. Comparison of survival analysis was performed using log-rank (Mantel-Cox) test; chi-square: 8.323, ***P=0.0039.
